# *Caenorhabditis elegans* Histone Methyltransferase MET-2 Shields the Male X Chromosome from Checkpoint Machinery and Mediates Meiotic Sex Chromosome Inactivation

**DOI:** 10.1371/journal.pgen.1002267

**Published:** 2011-09-01

**Authors:** Paula M. Checchi, JoAnne Engebrecht

**Affiliations:** Department of Molecular and Cellular Biology, University of California Davis, Davis, California, United States of America; Massachusetts General Hospital, Howard Hughes Medical Institute, United States of America

## Abstract

Meiosis is a specialized form of cellular division that results in the precise halving of the genome to produce gametes for sexual reproduction. Checkpoints function during meiosis to detect errors and subsequently to activate a signaling cascade that prevents the formation of aneuploid gametes. Indeed, asynapsis of a homologous chromosome pair elicits a checkpoint response that can in turn trigger germline apoptosis. In a heterogametic germ line, however, sex chromosomes proceed through meiosis with unsynapsed regions and are not recognized by checkpoint machinery. We conducted a directed RNAi screen in *Caenorhabditis elegans* to identify regulatory factors that prevent recognition of heteromorphic sex chromosomes as unpaired and uncovered a role for the SET domain histone H3 lysine 9 histone methyltransferase (HMTase) MET-2 and two additional HMTases in shielding the male X from checkpoint machinery. We found that MET-2 also mediates the transcriptional silencing program of meiotic sex chromosome inactivation (MSCI) but not meiotic silencing of unsynapsed chromatin (MSUC), suggesting that these processes are distinct. Further, MSCI and checkpoint shielding can be uncoupled, as double-strand breaks targeted to an unpaired, transcriptionally silenced extra-chromosomal array induce checkpoint activation in germ lines depleted for *met-2*. In summary, our data uncover a mechanism by which repressive chromatin architecture enables checkpoint proteins to distinguish between the partnerless male X chromosome and asynapsed chromosomes thereby shielding the lone X from inappropriate activation of an apoptotic program.

## Author Summary

Meiosis results in the generation of non-identical haploid gametes and maintenance of chromosome number during sexual reproduction. Precise meiotic chromosome segregation is essential for life, and in humans errors in this process contribute to aneuploidy or failure in meiosis, which manifests as spontaneous abortions or infertility. Cellular surveillance pathways monitor the steps of meiosis; and, if homologous chromosomes fail to pair and recombine, checkpoint machinery responds by eliciting signals to induce apoptosis. However, in many species males possess a single X chromosome that is transcriptionally silenced, accumulates repressive histone marks, and is not recognized as partnerless by meiotic checkpoints. Here, we used *C. elegans* to investigate how the male X is precluded from checkpoint signaling and uncovered a role for conserved chromatin-remodeling proteins that block checkpoints and mediate meiotic silencing. Our data elucidate the molecular mechanisms by which chromatin architecture influences both transcriptional silencing and checkpoint response to breaks on unpaired sex chromosomes, and we propose a model by which repressive chromatin modifiers directly block meiotic checkpoints from accessing the male X chromosome.

## Introduction

In sexually reproducing organisms, meiotic cell division is responsible for the faithful segregation of genetic material to sperm and egg [1]. Errors in this process contribute to aneuploidy and genomic instability and are an underlying cause of human reproductive problems [2]. A number of conserved checkpoint mechanisms monitor the steps of meiosis and respond to errors by eliciting signals to activate repair pathways and/or to induce germline apoptosis [2], [3]. The mechanisms by which meiotic checkpoint machinery responds to errors differ between males and females, and these differences are proposed to contribute to the high frequency of meiotic failure observed in human oocytes [4].

One distinction between female and male meiosis that may account for differences in checkpoint function is sex chromosome karyotype. Whereas human females possess a pair of homologous X chromosomes, males have a single, largely unpaired X and non-homologous Y, whose unpaired status is not recognized by checkpoint machinery [5]. While multiple strategies have evolved to promote the segregation of heteromorphic sex chromosomes [5]–[7], the molecular mechanisms responsible for shielding the sex chromosomes from meiotic checkpoints and preventing inappropriate apoptosis are unknown.

During mammalian male meiosis, the heteromorphic sex chromosomes undergo a silencing process called meiotic sex chromosome inactivation (MSCI), which results in the elaboration of a specialized chromatin domain and transcriptional silencing [8], [9]. In mice, defects in MSCI result in pachytene arrest and elevated germline apoptosis, presumably due to expression of sex-linked genes deleterious for male meiosis [10]. Interestingly, asynapsed regions of homologous chromosomes are also epigenetically marked by a related process termed meiotic silencing of unsynapsed chromatin (MSUC) [8], [11]. While MSCI correlates with the shielding of heteromorphic sex chromosomes from being recognized as unpaired by meiotic checkpoints, MSUC does not block recognition of unpaired homologous chromosomes, suggesting that MSUC and MSCI have distinct properties and outputs.

Although sex chromosomes have evolved independently many times, aspects of sex chromosome regulation have many common features [12]. As in humans, *Caeonorbaditis elegans* males have a single X chromosome that is subject to MSCI and is precluded from meiotic checkpoints [5], [13], [14]. Similar to the XY body during mammalian spermatogenesis [5], the lone X chromosome in male worms is highly condensed and accumulates the repressive histone modification dimethylation of histone H3 on lysine 9 (H3K9me2) [15]–[17]. Consistent with transcriptional silencing characteristic of MSCI, X-linked transcripts have not been detected by *in situ* hybridization in *C. elegans* heterogametic germ lines [13], [16], and microarray analyses have revealed a significant under-representation of sperm-expressed genes on the X [18].

To determine how heteromorphic sex chromosomes are shielded from recognition by checkpoints, we conducted a directed RNAi screen *in C. elegans* to identify germline-enriched chromatin modifiers that block checkpoint signaling and germline apoptosis in the heterogametic germ line. As males lack germline apoptosis [19], we utilized *fem-3(lf)* X0 worms, a sex determination mutant that has an oogenic germ line competent for checkpoint-activated apoptosis but a single, unpaired X chromosome [13], [20]. Similar to the male X, the single X in *fem-3(lf)* females accumulates repressive chromatin marks, is transcriptionally quiescent, and is not recognized by meiotic checkpoints as partnerless [13]. We identified a novel role for three conserved SET domain histone methyltransferases (HMTases), MES-2, MET-1 and MET-2, in this process. Here, we focus primarily upon understanding the function of *met-2*, which encodes the ortholog of mammalian protein SetDB1, a H3K9 HMTase that maintains transcriptional quiescence in embryonic stem cells [21], [22]. In *C. elegans*, MET-2 is the HMTase required for all H3K9me2 deposition in the adult germ line [23], and in this study we demonstrate that MET-2-dependent H3K9me2 has distinct outputs on the unpaired X chromosome versus asynapsed homologous chromosomes with respect to both checkpoint signaling and transcriptional silencing.

## Results

### A subset of chromatin modifiers inhibit apoptosis in the heterogametic germ line

In the *C. elegans* heterogametic (X0) germ line, the single, unpaired X chromosome is highly condensed, accumulates a subset of repressive histone modifications, and is not recognized by meiotic checkpoints [13], [15]–[17]. We therefore hypothesized that the unique heterochromatin architecture of the lone X chromosome directly prohibits access of checkpoint proteins that would otherwise recognize the unpaired X as problematic and trigger meiotic checkpoint activation. To this end, we individually depleted twenty-nine germline-enriched chromatin modifiers and identified candidates whose knockdown increased apoptosis in *fem-3(lf)* X0 germ lines versus wild-type (XX) germ lines expressing CED-1::GFP, a fusion protein that is expressed in sheath cells and marks early apoptotic corpses [24] (Table S1).

RNAi depletion of approximately one-third of the candidates screened resulted in an elevated number of CED-1::GFP(+) nuclei in both homogametic (XX) and heterogametic [*fem-3(lf)* X0] germ lines (Table S1), indicating a broad requirement for chromatin architecture in maintaining overall germline homeostasis throughout meiosis. We identified seven candidates whose absence resulted in significantly elevated apoptosis in heterogametic (X0) but not homogametic (XX) germ lines (Table S1). From these candidates, levels of X0-specific germline apoptosis were highest in the SET domain HMTases MES-2, MET-1, and MET-2 (Table 1). MES-2 is the homolog of Enhancer of zeste E(z), and in the *C. elegans* germ line, MES-2 promotes acquisition of the repressive marks H3K27me2/3 and H3K9me3 [23], [25]. MET-1 and MET-2 are homologs of the yeast H3K36 HMT Set2 and the mammalian H3K9 HMT SetDB1, respectively, and in *C. elegans*, they are required for transcriptional repression of *lin-3* during vulval development [21]. In the adult germ line, MET-2 is also required for accumulation of the repressive histone modification H3K9me2 and is suggested to play a role in ensuring the fidelity of chromosome segregation during meiotic progression [23]. Identification of these HMTases in our screen suggests a role for repressive histone modifications in shielding the X chromosome from being recognized as unpaired and triggering checkpoint activation.

**Table 1:** Absence of a subset of SET-domain HMTases results in elevated apoptosis in the heterogametic (X0) germ line.

		# Apoptotic bodies/gonad	
RNAi	Description/Relevant Function	wild-type XX	*fem-3(lf)* X0
L4440	N/A	6.5±0.1	2.5±0.1
*met-1*	Set2 homolog; H3K36 methylation	5.5±0.3	4.4±0.2*
*met-2*	SETDB1 homolog; H3K9 methylation	6.1±0.2	4.1±0.2*
*mes-2*	Enhancer of zeste (EZH2) ortholog; H3K27 methylation	7.0±0.4	4.7±0.2*

Apoptotic bodies were scored by quantifying CED-1::GFP expressing nuclei per gonad arm. L4 wild-type XX hermaphrodites or young adult *fem-3(lf)* X0 females expressing CED-1::GFP were transferred to RNAi feeding plates (see Materials and Methods) and scored after approx. 48 hrs. *fem-3(lf)* X0 germ lines have an overall lower level of apoptosis than wild-type XX germ lines, which is presumably a consequence of delayed meiotic progression due to the absence of sperm production [5], [13], [14]. Total number of gonads examined: N2 L4440 XX, *N* = 457; *met-1*(RNAi) XX, *N* = 55; *met-2*(RNAi) XX, N = 129; *mes-2*(RNAi) XX, *N* = 63; *fem-3(lf)* L4440 X0, *N* = 383; *fem-3(lf)*;*met-1*(RNAi) X0, *N* = 73; *fem-3(lf)*;*met-2*(RNAi) X0, *N* = 141; *fem-3(lf)*;*mes-2*(RNAi) X0, *N* = 82. Data shown are means ± S.E.M., and statistical comparisons between RNAi knockdown and empty L4440 vector were determined using a two-tailed Mann-Whitney test;

*: denotes p<0.0001. See Table S1 for entire data set from directed RNAi screen.

### Absence of *met-2* in X0 germ lines results in increased apoptosis by activating the recombination checkpoint

As H3K9me2 deposition is a conserved feature of heteromorphic sex chromosomes ([14], [26], [27]), we focused our experiments on *met-2*. RNAi knockdown of *met-2* resulted in abrogation of H3K9me2 staining and corresponded to elevated apoptosis exclusively in the X0 germ line (Figure S1; Table 1). XX animals did not show a similar dependence on *met-2* for apoptosis; the number of CED-1::GFP(+) nuclei was not significantly affected in either *met-2*(RNAi) XX or *met-2(n4256)* XX deletion mutant germ lines (Figure S2B). We also assessed germline apoptosis in *met-2(n4256)* XX mutants using acridine orange (AO) and obtained similar results (Figure S2C).

**Figure 1:**
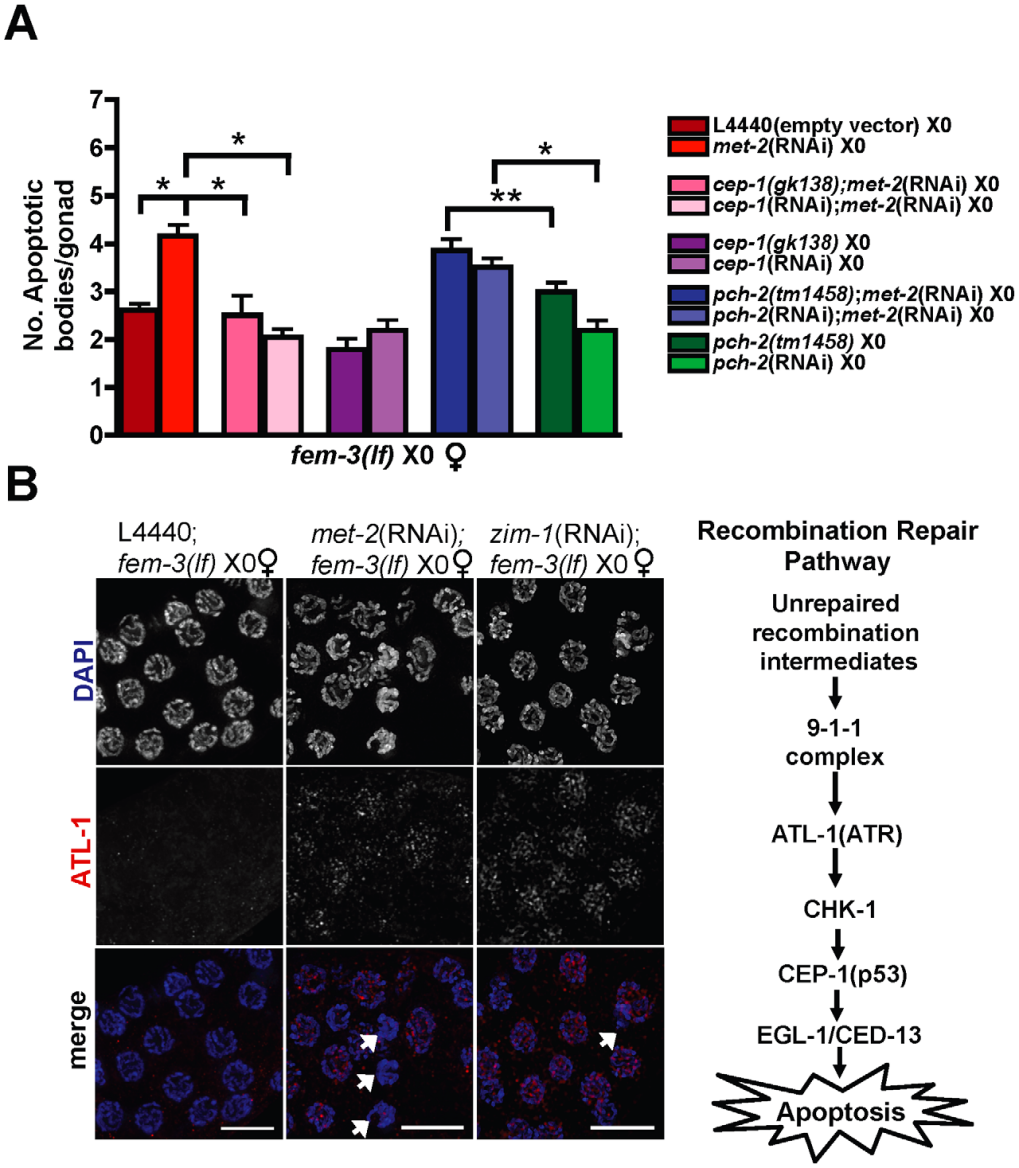
Absence of MET-2 triggers the recombination checkpoint in worms with a single X. (A) *fem-3(lf)* X0 germ lines expressing CED-1::GFP have elevated apoptosis when depleted for *met-2*, which is dependent upon *cep-1* but not *pch-2*. Apoptosis was scored in *fem-3(lf)* X0 adults approximately 48 hr post-L4. Total number of gonads examined: *fem-3(lf)* L4440, *N* = 116; *met-2*(RNAi); *fem-3(lf)*, *N* = 31; *met-2*(RNAi);*cep-1(gk138)*;*fem-3(lf) N* = 26; *met-2*(RNAi);*cep-1*(RNAi);*fem-3(lf)*, *N* = 46; *met-2*(RNAi);*pch-2(tm1458)*;*fem-3(lf) N* = 56; *met-2*(RNAi);*pch-2*(RNAi);*fem-3(lf) N* = 60; *pch-2(tm1458)*;*fem-3(lf) N* = 83; *pch*-2(RNAi);*fem-3(lf)*, *N* = 49; *cep-1(gk138)*;*fem-3(lf) N* = 44; *cep-1*(RNAi);*fem-3(lf) N* = 38. Statistical comparisons between data sets were conducted using a two-tailed Mann-Whitney test. * denotes p≤0.001, and ** denotes p≤0.05. Error bars correspond to S.E.M. (B) Right: Schematic of *C. elegans* recombination repair pathway. Left: Late pachytene *fem-3(lf)* X0 germ line nuclei stained with DAPI (blue) and ATL-1 (red). ATL-1 is not present in control *fem-3(lf)* X0 germ lines fed empty L4440 vector, whereas *met-2*(RNAi);*fem-3(lf)* X0 and *zim-1*(RNAi);*fem-3(lf)* X0 germ lines accumulate ATL-1 foci in pachytene nuclei. We did not observe any ATL-1 staining in *atl-1*(RNAi) germ lines, consistent with previous studies demonstrating specificity of this antibody (data not shown; [19]). White arrows denote apoptotic corpses. Scale bar = 10 µm.

To determine if the elevated level of apoptosis observed in the absence of *met-2* was the result of checkpoint activation, we scored apoptosis in *met-2*(RNAi); *fem-3(lf)* X0 germ lines depleted or mutant for meiotic checkpoint machinery. In *C. elegans*, meiotic progression is monitored by two distinct checkpoints that distinguish and respond to errors in synapsis or recombination defects [28]. To determine whether the synapsis checkpoint was activated, we scored apoptosis in the absence of the pachytene checkpoint protein PCH-2 (Pch2), which in *C. elegans* is triggered in response to unsynapsed chromosomal pairing centers [28]. We observed no decrease in apoptosis in the absence of *pch-2* [*pch-2*(RNAi);*met-2*(RNAi);*fem-3(lf)* X0 and *pch-2*(*tm1458*);*met-2*(RNAi);*fem-3(lf)* X0] compared to *met-2*(RNAi);*fem-3(lf)* X0 alone (Figure 1A), suggesting that *met-2*-dependent apoptosis is not activated by the synapsis checkpoint.

To test whether RNAi knockdown of *met-2* triggered the recombination checkpoint in response to meiotic DSBs that are induced on the single X [13], we scored the number of CED-1::GFP(+) nuclei in *met-2*(RNAi);*fem-3(lf)* X0 germ lines inactivated for *cep-1*, which encodes the ortholog of the human tumor suppressor p53 and is required for the detection of persistent recombination intermediates but not chromosome asynapsis in worms [28]–[30]. RNAi knockdown or a deletion mutant of *cep-1* [*cep-1*(RNAi);*met-2*(RNAi);*fem-3(lf)*X0 and *cep-1(gk138)*;*met-2*(RNAi);*fem-3(lf)* X0], resulted in basal levels of apoptosis (Figure 1A), suggesting that the absence of *met-2* activates the recombination checkpoint. We also monitored localization of ATL-1 (ATR), a component of the recombination checkpoint pathway upstream of p53 [31], in *met-2*(RNAi);*fem-3(lf)* X0 germ lines. In response to checkpoint activating conditions including chromosome asynapsis, ATL-1 is recruited to germline nuclei and accumulates on all chromosomes [19], [31]. While we observed no ATL-1 nuclear accumulation in control, *fem-3(lf)* X0 germ lines, RNAi depletion of *met-2* resulted in the appearance of ATL-1 foci in late pachytene nuclei, coincident with the timing of apoptotic body accumulation (Figure 1B and Figure S2A).

To determine if activation of the recombination checkpoint in the absence of MET-2 was a consequence of either altered processing or an increased number of meiotic DSBs on the male X chromosome, we monitored RAD-51 foci formation. In wild-type *C. elegans* germ lines, RAD-51 accumulation begins in the transition zone (e.g. leptotene/zygotene) and is most abundant in late transition zone/early pachytene male nuclei [13], [32], prior to an X-specific enrichment of H3K9me2 (Figure S3A). We observed no difference in the number of X-specific RAD-51 foci in wild-type versus *met-2* mutant germ lines, indicating that MET-2 does not affect the kinetics of DSB processing or repair (Figure S3B). We also monitored RAD-51 foci in germ lines depleted for *rad-54*, which is required for homologous recombination-mediated repair [33], and observed no difference in the number of RAD-51 foci in *rad-54*(RNAi) versus *met-2*;*rad-54*(RNAi) nuclei (Figure S3C), suggesting that the absence of *met-2* does not result in recombination checkpoint activation due to an increased number of breaks on the X chromosome.

### Absence of repressive chromatin modifiers results in ectopic acquisition of H3K4me2 on the single X chromosome

**Figure 2:**
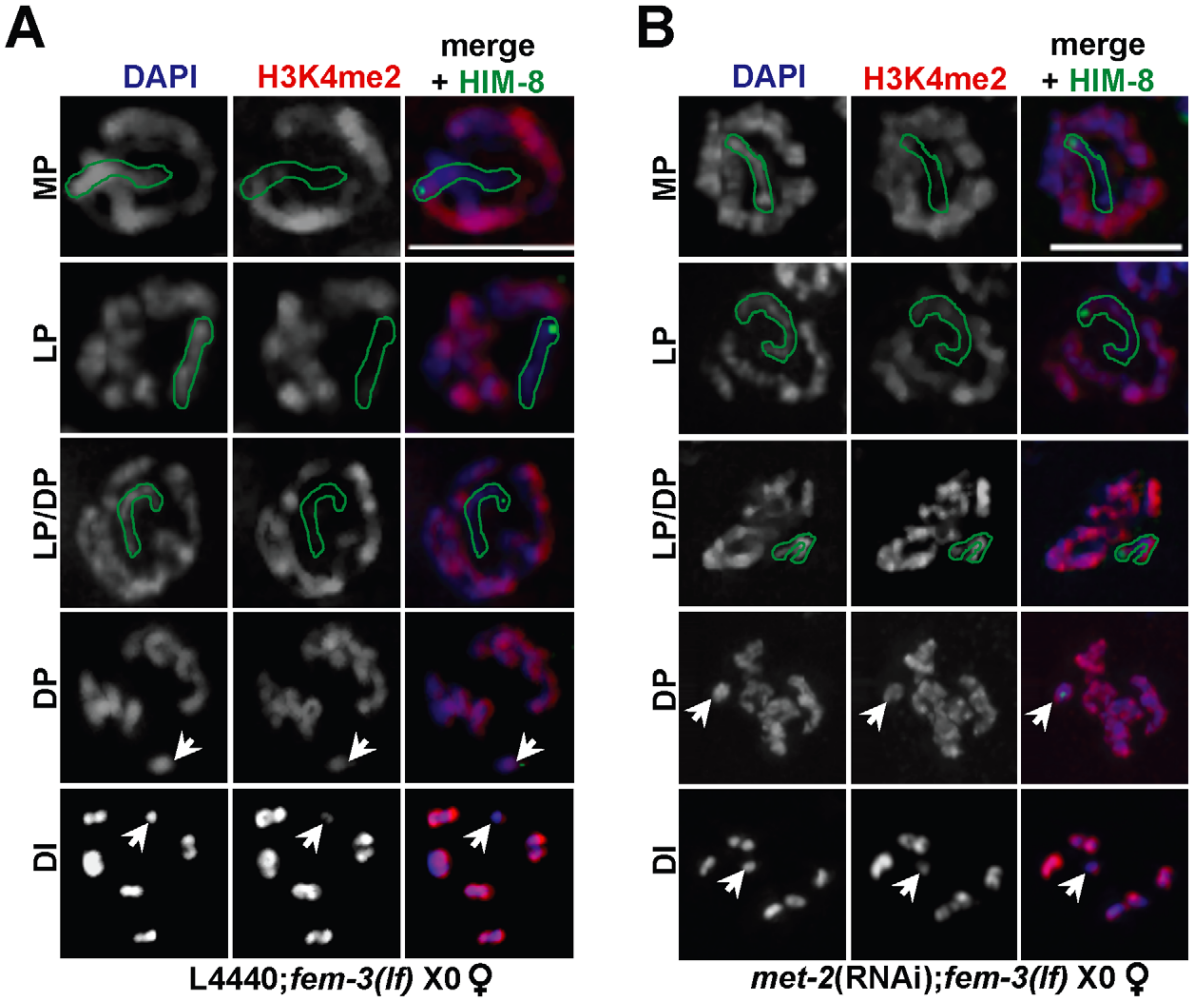
Ectopic X-specific H3K4me2 accumulation in late pachytene X0 germ lines depleted for *met-2*. Immunolocalization of H3K4me2 (red) counterstained with DAPI (blue) in *fem-3(lf)* X0 germ lines fed (A) empty L4440 vector (left) or (B) *met-2* dsRNA (right). Green outline indicates the X chromosome in MP, LP, and LP/DP, as determined by HIM-8 staining (green). White arrows indicate the X chromosome in DP and DI. Mid-pachytene (MP); Late pachytene (LP); diplotene (DP); diakinesis (DI). Scale bar = 5 µm. (See also Figure S4).

In heterogametic (X0) pachytene-stage germ lines, the single, unpaired X accumulates the repressive mark H3K9me2 and lacks the histone modification H3K4me2 [13], [16], [17], a mark corresponding to transcriptionally competent chromatin. In contrast to XX germ lines, which accumulate H3K4me2 on the X chromosome pair in late pachytene/early diplotene coincident with a transient accumulation of elongating RNA Polymerase II [34], X0 germ lines remain devoid of X-specific H3K4me2 until late diplotene [13], [15], [17], and H3K4me2 intensity on the single X is never as high as it is on the autosomes in diakinesis (Figure 2A). To test whether the absence of H3K9me2 altered the dynamics of X-specific H3K4me2 accumulation, we assessed H3K4me2 staining in *fem-3(lf)* X0 germ lines depleted for *met-2*. While H3K4me2 localization was unaffected in transition zone nuclei through mid-pachytene stage germ lines, we observed an ectopic accumulation of this mark on the single, unpaired X chromosome in late pachytene (Figure 2B), indicating that the absence of *met-2* alters the dynamics of other histone marks in addition to H3K9me2 on the partnerless X chromosome.

The other HMTases identified in our screen methylate distinct histone lysine residues: MES-2 promotes the acquisition of H3K27me2/3 and H3K9me3 [23], [25] while MET-1 methylates H3K36 [21], [35]. Consistent with this, H3K9me2 levels appear normal in the absence of either *mes-2* [23] or *met-1* (Figure S1). To determine whether the absence of these HMTases also resulted in ectopic X chromosome-specific H3K4me2 accumulation, we monitored H3K4me2 in *fem-3(lf)* X0 germ lines depleted for *mes-2* and *met-1*, and indeed, both *met-1*(RNAi); *fem-3(lf)* X0 and *mes*-*2*(RNAi); *fem-3(lf)* X0 germ lines exhibited ectopic H3K4me2 accumulation on the single, unpaired X in late pachytene (Figure S4). Together, these data suggest that multiple repressive marks are required to block recognition of the single, unpaired X by checkpoint machinery and that absence of any one of these repressive marks results in ectopic accumulation of H3K4me2 on the single X.

### MET-2 inhibits transcription of the single X

**Figure 3:**
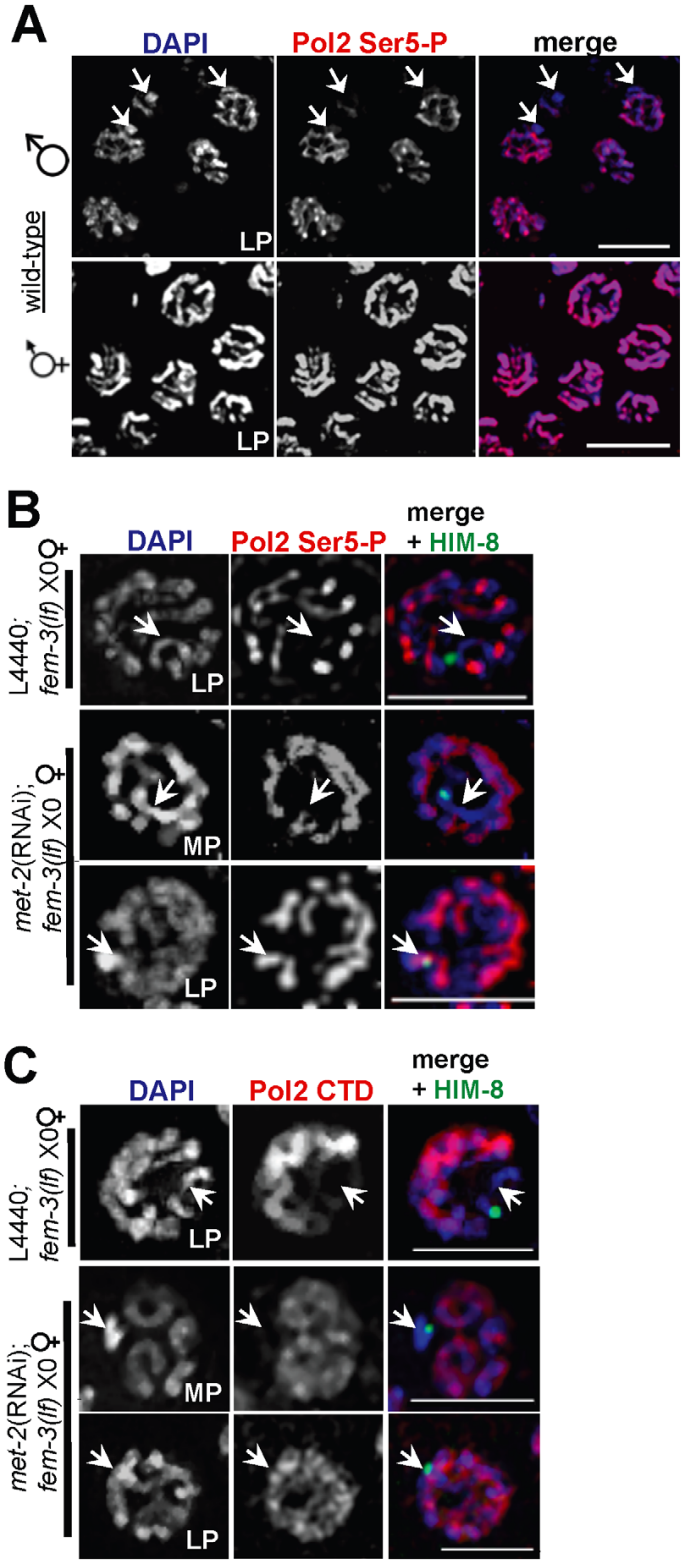
X chromosome-specific transcriptional regulation is specified by chromatin architecture and is disrupted in *met-2(RNAi)*;*fem-3(lf)* X0 germ lines. (A) Late pachytene stage nuclei from wild-type male X0 (top) and hermaphrodite XX (bottom) germ lines stained with activated Pol2 Ser5-P (red), and counterstained with DAPI (blue). White arrowheads (top) correspond to the unpaired X chromosome. Scale bar = 10 µm. (B) In *fem-3(lf)* X0 germ lines, Pol2 Ser5-P (red) is absent from the X chromosome (marked by white arrowheads and the X chromosome marker HIM-8 [40] in green) but appears on the single X in late pachytene in the absence of *met-2*. Scale bar = 5 µm. (C) The unphosphorylated form of Pol2 (CTD, red) is absent from the X chromosome (denoted by white arrowheads and HIM-8, green) in control *fem-3(lf)* X0 germ lines (top), but appears on the X in *met-2*(RNAi);*fem-3(lf)* X0 germ lines in late pachytene. Mid-pachytene (MP); Late pachytene (LP). Scale bar = 5 µm.

A hallmark of heteromorphic sex chromosomes is the acquisition of repressive histone modifications and transcriptional silencing. Because *met-2* affects the dynamics of H3K9me2 and H3K4me2, we hypothesized that the transcriptional status of the single X chromosome was also disrupted in heterogametic germ lines depleted for *met-2*. To examine transcriptional activity on the X, we used an antibody raised against the Ser5 phospho-epitope of the RNA Polymerase II C terminal domain (Pol2 Ser5-P) that localizes to transcriptionally competent chromatin [15], [36], [37]. As expected, Pol2 Ser5-P localized to chromatin in both sexes and remained visible until mid/late diplotene (Figure 3A; data not shown). Notably, while late pachytene stage hermaphrodite (XX) germ lines contained robust Pol2 Ser5-P staining on all chromosomes, all male germ line nuclei examined possessed a single, highly condensed chromosome devoid of Pol2 Ser5-P staining, which based on its morphology [38], we presume to be the X (Figure 3A).

To determine whether the presence of MET-2 is sufficient to block transcriptional activation, we examined the pattern of Pol2 Ser5-P in *fem-3(lf)* X0 germ lines depleted for *met-2*. In pre-meiotic germ cells through mid-pachytene, both control *fem-3(lf)* X0 and *met-2*(RNAi);*fem-3(lf)* X0 germ lines had identical patterns of Pol2 Ser5-P accumulation (data not shown). However, while Pol2 Ser5-P remained absent from the X chromosome throughout meiotic prophase in *fem-3(lf)* X0 germ lines, ectopic Pol2 Ser5-P accumulation was detected on the X chromosome in late pachytene *met-2*(RNAi);*fem-3(lf)* X0 germ lines (Figure 3B), indicating that MET-2 inhibits transcription from the single X chromosome.

To determine whether MET-2 inhibits transcription at the level of pre-initiation complex assembly, we used an antibody that recognizes the unphosphorylated form of Pol2 CTD, which is assembled at promoters prior to transcription initiation [39]. In control *fem-3(lf)* X0 germ lines, Pol2 CTD accumulation was identical to the pattern of Pol2 Ser5-P accumulation, and was detected abundantly on the autosomes from the distal tip (mitotically-dividing germline nuclei) through diplotene stage nuclei, yet was absent from the X chromosome in all stages examined (Figure 3C; data not shown). In *met-2*(RNAi);*fem-3(lf)* X0 germ lines, however, we observed ectopic, X-specific accumulation of Pol2 CTD in late pachytene, consistent with the timing of Pol2 Ser5-P appearance in these germ lines (Figure 3C).

**Figure 4:**
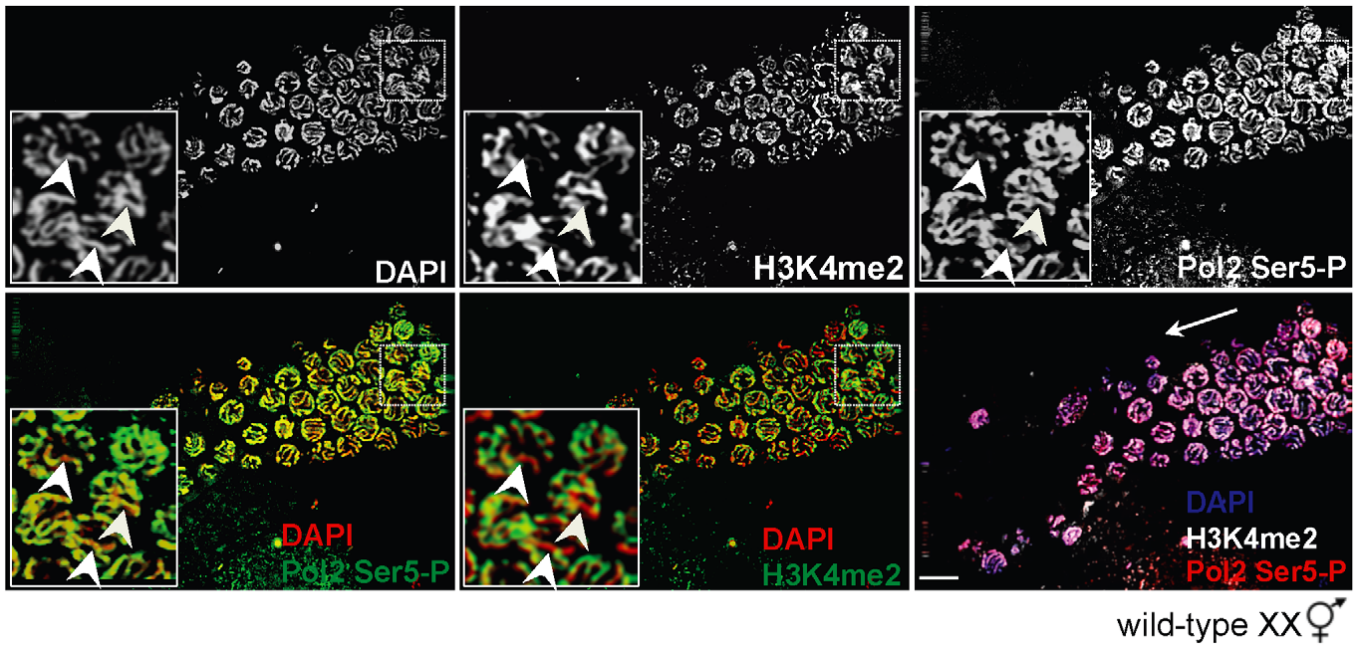
H3K4me2 is not required for Pol2 loading or promoter clearance on paired X chromosomes. Wild-type XX germ line stained for H3K4me2 (top, middle panel) and Pol2 Ser5-P (top, right panel) and counterstained with DAPI (top, left panel). In mid to late pachytene stage nuclei (inset), H3K4me2 (green, middle panels) is present on all chromosome pairs except the X (bottom, middle panel), whereas Pol2 Ser5-P (green, left panels) is present on all chromosomes (bottom left). Germ lines were counterstained with DAPI (red; blue in merge). Inset: White arrowheads denote the X chromosome pair. Merged image (bottom, right panel) shows DAPI (blue), H3K4me2 (white) and Pol2 Ser5-P (red). White arrow indicates direction of meiotic progression. Scale bar = 10 µm.

As the absence of *met-2* resulted in loss of H3K9me2, accumulation of H3K4me2, and transcriptional activation, we tested whether acquisition of H3K4me2 is required for Pol2 loading and promoter clearance. To this end, we examined Pol2 Ser5-P accumulation in wild-type XX germ lines co-stained with anti-H3K4me2. In mid-pachytene XX germ lines, only the sex chromosomes are devoid of H3K4me2 staining [15], making it possible to easily identify the X chromosome pair at this stage. Interestingly, despite the absence of X-chromosome specific H3K4me2 staining in mid-pachytene, we detected Pol2 Ser5-P staining on both the autosomes and the sex chromosomes of wild-type XX germ lines (Figure 4), suggesting that H3K4me2 accumulation is not an absolute requirement for transcriptional activation.

### H3K9me2 is required to repress checkpoint signaling and transcription during MSCI but not MSUC

**Figure 5:**
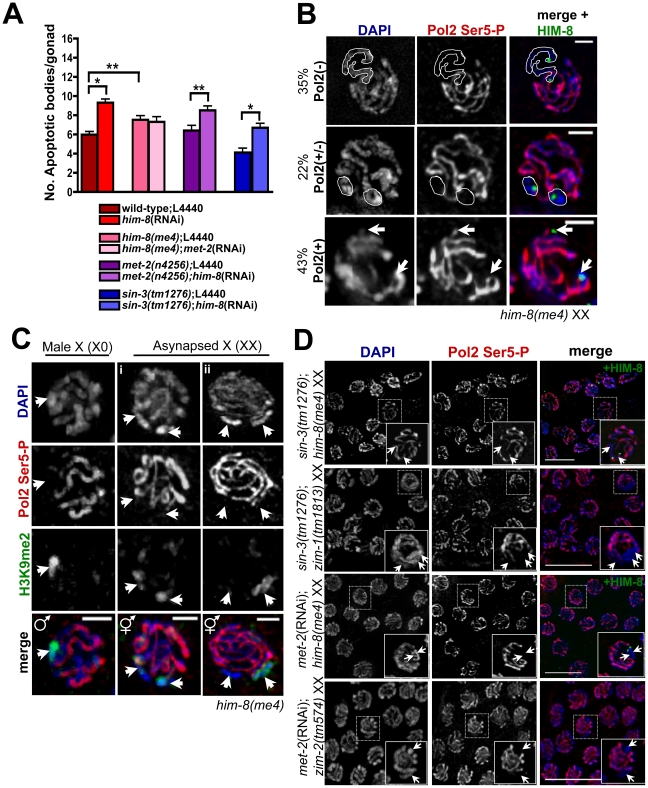
Germline apoptosis and Pol2 activation are not inhibited by H3K9me2 on asynapsed chromosome pairs. (A) Number of apoptotic bodies in XX germ lines as determined by CED-1::GFP fluorescence approx. 48 hr post L4 stage. Total number of gonads examined: N2 L4440 XX, *N* = 61; *him-8*(RNAi) XX, *N* = 74; *him-8(me4)* L4440, *N* = 54; *him-8(me4)*;*met-2*(RNAi), *N* = 26; *met-2(n4256)* L4440, *N* = 25; *met-2(n4256)*;*him-8*(RNAi), *N* = 33; *sin-3(tm1276)*, *N* = 18; *sin-3(tm1276)*;*him-8*(RNAi), *N* = 25. Statistical comparisons between data sets were conducted using a two-tailed Mann-Whitney test. * denotes p≤0.001, and ** denotes p≤0.05. Error bars correspond to S.E.M. (B) In *him-8(me4)* XX germ lines, three Pol2 Ser5-P (red) staining patterns were observed on asynapsed X chromosome pairs (identified by HIM-8 [green] and indicated by either white outlines [top and middle rows] or arrowheads [bottom rows]. We assessed Pol2 Ser5-P staining in pachytene nuclei from four *him-8(me4)* germ lines, and in 42/121 nuclei, Pol2 Ser5-P was missing throughout the length of the asynapsed X chromosomes [Pol2(−), top row]. 27/121 nuclei only lacked Pol2 Ser5-P on discrete regions of asynapsed X chromosomes adjacent to and containing the X chromosome pairing center [Pol2(+/−), middle row], and 52/121 nuclei contained Pol2 Ser5-P throughout the length of the asynapsed chromosome pairs [Pol2(+), bottom row]. Germ lines were counterstained with DAPI (blue). Scale bar = 2 µm. (C) The *him-8(me4)* male X chromosome (left) and most (30/35) *him-8(me4)* asynapsed X chromosome pairs (middle, i) were devoid of Pol2 Ser5 staining (red) and accumulated H3K9me2 staining (green) corresponding to unpaired DAPI-staining bodies (indicated by white arrowheads). Some (5/35) late pachytene *him-8(me4)* XX nuclei (right, ii) contained only one H3K9me2-enriched DAPI-staining body (green, arrowhead on right), while both asynapsed chromosomes were devoid of Pol2 Ser5-P staining (red). (D) *sin-3(tm1276)*;*him-8(me4)*, *sin-3(tm1276)*;*zim-1(1813)*, *met-2*(RNAi);*him-8(me4)*, and *met-2*(RNAi);*zim-2(574)* XX late pachytene germ line nuclei stained with Pol2 Ser5-P (red) and counterstained with DAPI (blue). In *him-8(me4)* XX germ lines, the asynapsed chromosomes were identified by co-staining with HIM-8 (green). Arrows indicate asynapsed chromosome pairs in inset. Scale bar = 10 µm.

In addition to the partnerless X, H3K9me2 accumulates on asynapsed chromosomes (both autosomes and sex chromosomes) [13], [16]; however, unlike the male X, asynapsed homologous chromosomes are recognized as unpaired and trigger checkpoint activation [40]. Given that MET-2-dependent H3K9me2 deposition on a single unpaired X chromosome protects germ lines from increased apoptosis (Table 1; Figure S2A–S2B), we asked whether MET-2 plays a general role in modulating apoptosis in the presence of asynapsed X chromosomes. To that end, we monitored apoptosis upon inactivation of *him-8*, which is required for pairing and synapsis of the X chromosome pair [40], in the presence or absence of *met-2* [*met-2*(RNAi);*him-8(me4)* XX and *him-8*(RNAi);*met-2(n4256)* XX strains expressing CED-1::GFP]. Surprisingly, while absence of *met-2* abrogates H3K9me2 enrichment on asynapsed X chromosomes [23], there was no corresponding effect on the number of apoptotic bodies in XX germ lines defective for X chromosome pairing (Figure 5A), suggesting that H3K9me2 deposition on an asynapsed chromosome pair does not inhibit checkpoint-activated germline apoptosis.

The apparent disconnect between the role of H3K9me2 deposition on the lone X chromosome (X0) versus asynapsed chromosomes (*him-8* XX) suggests that this modification may elicit a different functional response depending on the presence of a homologous partner. It has been proposed that multiple pathways exist to target H3K9me2 in the *C. elegans* germ line [41], and we hypothesized that an alternative pathway may be involved in either targeting or responding to H3K9me2 on asynapsed chromosomes. *sin-3* encodes a conserved histone deacetylase [42] that has been suggested to play a role in targeting H3K9me2 to unpaired chromatin in both mammals and worms [14], [41], [43]. Analysis of *sin-3* mutants revealed that in contrast to MET-2, SIN-3-dependent H3K9me2 deposition was specific for asynapsed chromosome pairs but not sequences lacking a pairing partner, as both the male X and a repetitive, extra-chromosomal array, *oxEx229* [44] retained H3K9me2 in the absence of SIN-3 (Figure S5A; data not shown). On the other hand, H3K9me2 acquisition on the asynapsed X chromosomes in *him-8* germ lines was dependent on both MET-2 and SIN-3 ([23]; Figure S5B and data not shown). These data suggest that SIN-3 is specific for H3K9me2 deposition on asynapsed chromosomes.

To determine whether SIN-3's role in monitoring chromosome pairing and H3K9me2 enrichment could be required to regulate checkpoint signaling in response to asynapsed chromosomes, we examined *sin-3(tm1276)* XX germ lines expressing CED-1::GFP and assessed germline apoptosis in this mutant as well as in response to depletion of *him-8*. In germ lines lacking SIN-3, we observed no difference in apoptosis compared to wild-type germ lines (Figure 5A). Further, while absence of *sin-3* eliminated H3K9me2 on the asynapsed X chromosome pair of *him-8(me4)* germ lines (Figure S5B), apoptosis was not affected (Figure 5A), indicating that SIN-3-dependent H3K9me2 deposition on asynapsed chromosomes does not affect checkpoint signaling.

To examine the transcriptional status of asynapsed chromosomes, we analyzed the loading/activation of Pol2 in *him-8* mutants. *him-8(me4)* encodes a missense mutation that results in defective X chromosome synapsis yet still generates a truncated protein product recognized by HIM-8 antibodies, allowing us to identify the X [40]. In contrast to male germ lines, which were completely devoid of Pol2 Ser5-P on the single, unpaired X chromosome (Figure 3A and Figure 5C), analysis of *him-8* revealed that Pol2 Ser5-P exhibited variable staining patterns: 35% of mid to late pachytene germ lines were devoid of Pol2 Ser5-P on the Xs and 43% contained Pol2 Ser5-P on all chromosomes (Figure 5B). The remaining 22% contained what appear to be only discrete X chromosome regions of Pol2 Ser5-P staining; in these examples we could identify regions surrounding the pairing center that lacked this epitope, yet Pol2 Ser5-P staining did not appear to be missing throughout the length of the unpaired chromosomes (Figure 5B; data not shown). We also monitored Pol2 Ser5-P in *zim-1*, in which chromosomes II and III are asynapsed and *zim-2*, in which chromosome V pairs are asynapsed [40], and we observed the same variable staining pattern (Figure S6A; data not shown). Together, these results suggest that while transcription is inhibited on asynapsed chromosomes, it is not completely blocked as occurs on the single X.

To probe the relationship between H3K9me2 deposition and transcriptional silencing on the single X versus the asynapsed X chromosome pair, we monitored H3K9me2 and Pol2 Ser5-P in *him-8* mutant male and hermaphrodite mid to late pachytene stage germ cells. Consistent with our previous results, we saw robust H3K9me2 staining and an absence of Pol2 Ser5-P on the single X throughout pachytene in male germ lines (Figure 5C, left panel; data not shown). In *him-8* XX germ lines, H3K9me2 staining on the asynapsed X chromosomes was less robust yet these chromosomes lacked Pol2 Ser5-P (Figure 5Ci). However, we occasionally observed mid-pachytene nuclei containing only a single H3K9me2 staining body even though Pol2 Ser5-P was absent from two chromosomes (Figure 5Cii). Additionally, as previously demonstrated for wild-type hermaphrodite germ lines [15], we also observed H3K9me2 accumulation on the autosomes in late pachytene *him-8* XX nuclei; at this stage, there was no apparent correlation between H3K9me2 deposition and Pol2 Ser5-P staining (Figure S6B; data not shown). These results suggest that unlike the male X, there is not a strict correlation between H3K9me2 deposition and transcriptional repression on asynapsed chromosomes.

The observation that transcriptional repression can occur in the absence of H3K9me2 on asynapsed chromosomes suggests that MET-2 and SIN-3 are not required for transcriptional silencing during MSUC. To examine the consequence of inactivating MET-2 and SIN-3 on transcription in the presence of asynapsed chromosomes, we monitored Pol2 Ser5-P in *sin-3(tm1276)* and *met-2*-depleted germ lines [*sin-3(tm1276)*;*him-8(me4), sin-3(tm1276)*;*zim-1(tm1813)*, *met-2*(RNAi);*him-8(me4)*, and *met*-2(RNAi);*zim-2(tm574)*]. Consistent with our findings above, Pol2 Ser5-P staining in *sin-3(tm1276)*;*him-8(me4)* and *sin-3(tm1276)*;*zim-1(tm1813)* double mutants was identical to that of *him-8(me4)* and *zim-1(tm1813)* XX germ lines, indicating that SIN-3 had no effect on the transcriptional status of asynapsed chromosomes (Figure 5D and Figure S6A). Similarly, in *him-8(me4)* and *zim-2(tm574)* germ lines depleted for *met-2*, Pol2 Ser5-P staining was unaffected compared to either mutant alone (Figure 5D and Figure S6). These data suggest that unlike the situation on the lone X, there is not a direct relationship between H3K9me2 and Pol2 activity on asynapsed chromosomes, and that H3K9me2 has different outputs on sequences lacking a pairing partner versus asynapsed chromosome pairs.

### A repressive chromatin environment is sufficient to block checkpoint signaling in response to targeted DSBs in the absence of a homologous partner

**Figure 6:**
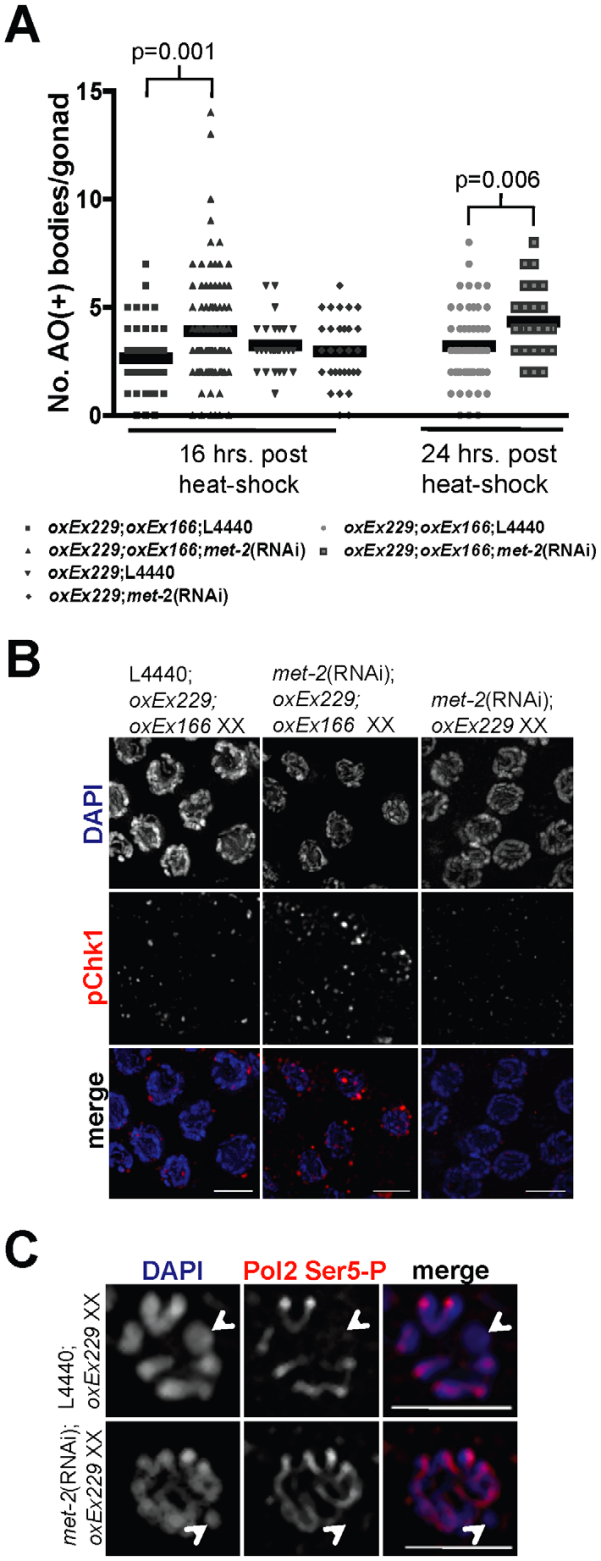
In the absence of MET-2, DSBs targeted to an unpaired extra-chromosomal array induces checkpoint-dependent apoptosis independent of transcriptional activation. (A) Scatterplot depicting number of apoptotic bodies per gonad arm as detected by AO staining at 16 and 24 hr following heat-shock in wild-type N2 *oxEx166*;*oxEx229*;L4440 XX germ lines (squares = 16 hr; circles = 24 hrs), *oxEx166*;*oxEx229*;*met-2*(RNAi) XX germ lines (upward-pointing triangles = 16 hr; outlined squares = 24 hr), N2 *oxEx229*;L4440 XX germ lines (downward-pointing triangles), and *met-2*(RNAi);*oxEx229* XX germ lines (diamonds). Horizontal black lines correspond to mean for each data set. Total number of gonads examined: 16 hr: N2 *oxEx166*;*oxEx229* XX, *N* = 45; *met-2*(RNAi);*oxEx166*;*oxEx229* XX, *N* = 102; N2 *oxEx229* XX, *N* = 30; *met-2*(RNAi);*oxEx229* XX, *N* = 31. 24 hr: N2 *oxEx166*;*oxEx229* XX, *N* = 61; *met-2*(RNAi);*oxEx166*;*oxEx229* XX, *N* = 26. Statistical comparisons between data sets were conducted using a two-tailed Mann-Whitney test, and p values between statistically significant data sets are indicated above corresponding data points. (B) Late pachytene *oxEx166*;*oxEx229*;L4440 XX, *oxEx166*;*oxEx229*;*met-2*(RNAi) XX, and *oxEx229*;*met-2*(RNAi) XX germ line nuclei stained with phospho-Chk1(Ser345) (pChk1) (red) and counterstained with DAPI (blue). Germ line dissections and immunostaining were performed approximately 16 hr following heat-shock. pChk1 specificity was determined using *chk-1* RNAi (date not shown) and was consistent with results in [19]. Scale bar = 5 µm. (C) Late pachytene/diplotene stage nuclei from control *oxEx229*;L4440 (top) and *met-2*(RNAi);*oxEx229* germ lines stained with Pol2 Ser5-P (red) and counterstained with DAPI (blue). White arrowheads indicate extra-chromosomal array (*oxEx229*). Scale bar = 5 µm.

DSB accumulation and processing are distinct between paired chromosomes and a single, unpaired X chromosome, indicating that dynamics of DSB repair are likely to be influenced by chromatin architecture as well as the presence of a homologous pairing partner [13]. To investigate how chromatin architecture and lack of a pairing partner influence response to DSBs, we used *Mos1* mutagenesis to induce targeted breaks to unpaired DNA in the presence or absence of repressive chromatin marks. We hypothesized that *oxEx229*, a repetitive extra-chromosomal array that contains multiple copies of the *Mos1* substrate [44], is recognized in a manner similar to that of the single X chromosome. Analogous to the lone X, the *oxEx229* array lacks a homologous pairing partner and is modified by MET-2, but not SIN-3 (Figures S5A and S7A–S7B). We therefore tested whether DSBs targeted to *oxEx229* resulted in increased accessibility to checkpoint proteins and elevated apoptosis in the absence of *met-2*. We generated double-transgenic worms containing both *oxEx229* as well as *oxEx166* (an array containing a heat-shock inducible *Mos1* transposase) and monitored apoptosis in heat-shocked worms grown in the presence or absence of *met-2* dsRNA. As a control, we scored apoptosis in heat-shocked germ lines containing only the *Mos1* substrate array (*oxEx229+*;*oxEx166−)*; in these germ lines, absence of *met-2* had no effect, indicating that any differences observed were specific to the *Mos1*-induced DSBs. In heat-shocked, *oxEx229+*;*oxEx166+* germ lines depleted for *met-2*, we observed an increase in apoptosis as measured by AO staining compared to controls (Figure 6A, p = 0.001).

To determine whether targeted DSBs in *oxEx229+*;*oxEx166+* germ lines depleted for *met-2* recruit meiotic checkpoint proteins, we stained heat-shocked germ lines with an antibody against activated phosphorylated Chk1 (pChk1). In response to either damage or errors in meiosis, pChk1 is recruited to germline nuclei and accumulates on all chromatin similarly to ATL-1 [19]. We observed an accumulation of pChk1 foci in pachytene-stage *met-2*(RNAi); *oxEx229+*;*oxEx166+* germ lines in response to *Mos1* transposase activation compared to control (empty L4440) or *met-2*(RNAi);*oxEx229* (*Mos1* substrate-only) germ lines (Figure 6B), demonstrating DSBs targeted to an extra-chromosomal array induce checkpoint-dependent apoptosis in germ lines depleted for *met-2*. Together, these results suggest that the absence of MET-2-dependent H3K9me2 creates a chromatin environment conducive to checkpoint signaling in response to a break on sequences lacking a partner.

**Figure 7:**
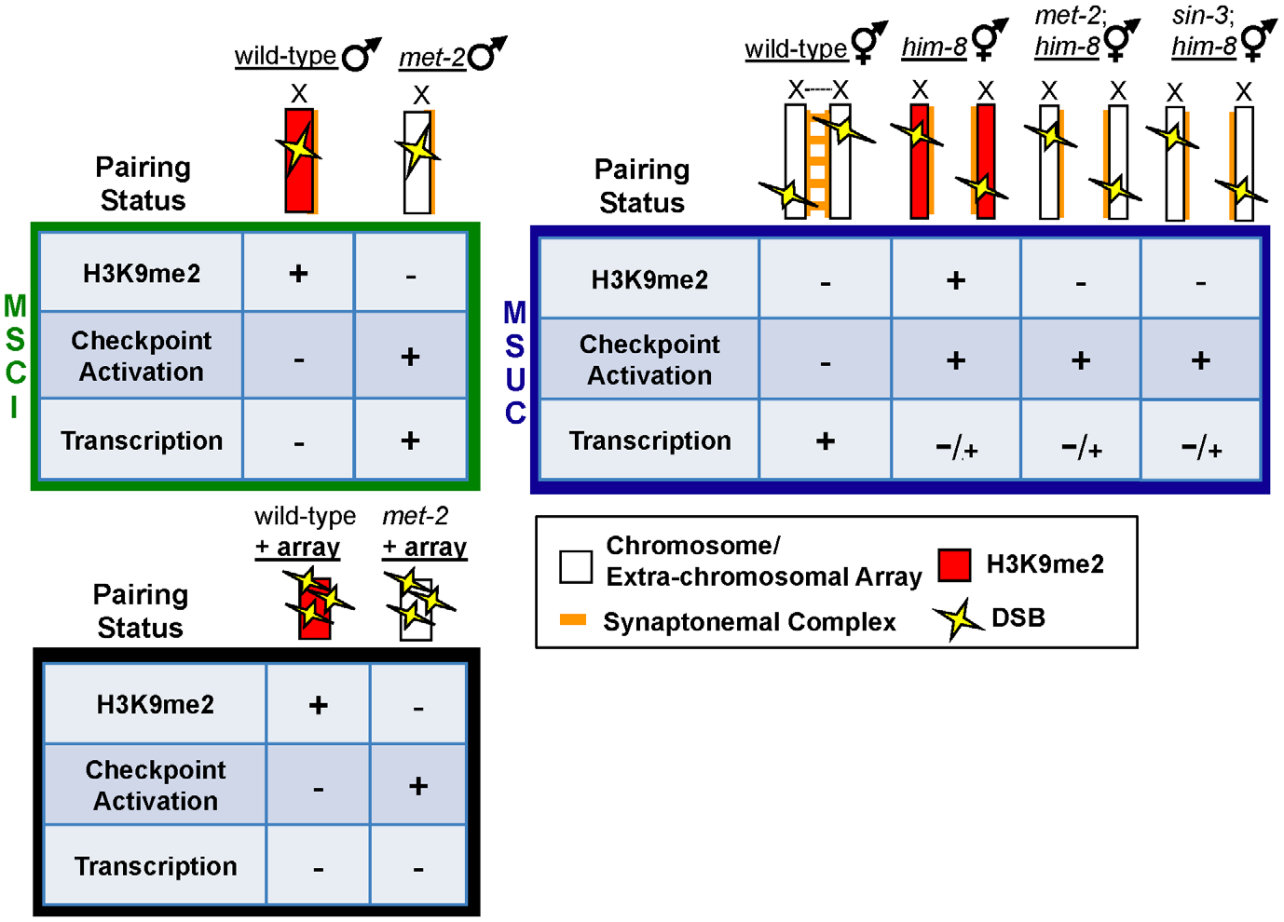
Repressive chromatin architecture blocks meiotic checkpoint signaling and facilitates MSCI on the single X chromosome but is dispensable for transcriptional inactivation on asynapsed chromosome pairs. During MSCI (top left, green box), a single, unpaired, and heterochromatinized X chromosome contains repressive chromatin marks including H3K9me2 (red) that corresponds to transcriptional inactivation and blocks meiotic checkpoint activation, even in the presence of DSBs (yellow lightening bolts). In heterogametic *met-2* germ lines, the single, unpaired X chromosome lacks H3K9me2, and DSBs on the lone X activate meiotic checkpoints. In response to DSBs targeted to an extra-chromosomal array (bottom, black box), absence of MET-2 results in checkpoint activation, while the array remains transcriptionally silenced. During MSUC (top right, blue box) chromosome asynapsis corresponds to transcriptional inactivation and H3K9me2 deposition on unpaired DNA, but unlike the male X chromosome, asynapsed X chromosomes elicit a checkpoint response. H3K9me2 deposition on asynapsed chromosome pairs is targeted by chromatin remodelers MET-2 and SIN-3; however, absence of either protein fails to re-establish Pol2 activation in response to pairing defects.

Our analyses indicate that on the single, unpaired X chromosome, absence of MET-2 results not only in loss of H3K9me2, but also acquisition of H3K4me2 and transcription in late pachytene germ lines, coincident with checkpoint activation and elevated apoptosis (Figure 1, Figure 2, Figure 3, and Figure S2). To determine the status of H3K4me2 and transcription on the extra-chromosomal array, we stained germ lines depleted for *met-2* and found that neither H3K4me2 nor the activated form of Pol2 were observed on the array (Figure S7A–S7B and Figure 6C). These data indicate that the role of MET-2 in repressing checkpoint signaling can be uncoupled from its role in transcriptional silencing: DSBs targeted to the *oxEx229* array lacking H3K9me2 results in checkpoint signaling in the absence of transcription (Figure 6 and Figure 7).

Another property of many extra-chromosomal arrays, yet not the single X chromosome, is enrichment for H3K9me3, a mark that is independent of H3K9me2 in the *C. elegan*s germ line, yet is also associated with heterochromatin and transcriptional silencing [23]. To investigate whether the *oxEx229* array is subject to H3K9me3 enrichment, we examined this mark in both control and *met-2*(RNAi) germ lines carrying the array. As expected, the pattern of H3K9me3 staining was identical in both control and *met-2*(RNAi) germ lines (see [23]) and was detected on all chromosomes throughout meiotic prophase (data not shown). Notably, we observed a robust enrichment of H3K9me3 accumulation on *oxEx229* from late pachytene through diakinesis (Figure S7C), which could potentially account for the corresponding absence of Pol2 observed in both control germ lines as well as those lacking *met-2* (Figure 6C). In summary, these results corroborate previous studies proposing independent roles for H3K9me2 and H3K9me3 enrichment, and demonstrate that the presence of H3K9me2 is sufficient to block checkpoint-dependent apoptosis in response to unpaired DNA.

## Discussion

The presence of unpaired (or partially paired) sex chromosomes presents a unique challenge during meiosis in the heterogametic sex. Consequently, multiple strategies have evolved to handle and segregate heterologous sex chromosomes while concurrently preventing their unpaired status from being recognized as problematic and hence activating meiotic checkpoints. Here, we have identified MET-2 as being required to shield the partnerless X chromosome from checkpoints and to mediate MSCI. As in mammals, heterochromatinization of the *C. elegans* male X chromosome is associated with repression of X-linked gene expression, which we have demonstrated is due to failure to load Pol2. Our analyses indicate that these processes, while interrelated, can be uncoupled and suggest that the unique chromatin environment of sex chromosomes safeguards gamete production in the heterogametic sex.

### Distinct targeting and outputs of H3K9me2 deposition on partnerless sex chromosomes versus asynapsed homologous chromosomes

In this study we provide evidence that the epigenetic landscape of sex chromosomes directly modulates meiotic checkpoint signaling, supporting a model whereby the chromatin architecture of the male X chromosome prevents triggering of a checkpoint in response to breaks ([6]; Figure 7). We identified a role for the HMTase MET-2 in blocking checkpoint signaling and mediating MSCI on the single X, as *fem-3(lf)* X0 germ lines depleted for *met-2* have elevated checkpoint-dependent apoptosis and load and activate Pol2. Previous reports have suggested that heterochromatization of the X chromosome depends on the absence of a pairing partner [17], and consequently, differences in sex chromosome karyotype may prevent activation of a checkpoint that detects chromosome asynapsis. Interestingly, our data suggest that meiotic checkpoint machinery is capable of distinguishing between asynapsed chromosomes and a single unpaired X, even though MET-2-dependent H3K9me2 modifies both. The mechanism by which the recombination checkpoint distinguishes between asynapsed chromosomes and a single unpaired X is not clear. One possibility is that there is a counting mechanism to distinguish sex chromosome karyotype and elicit the appropriate response. During development the dosage compensation complex is controlled by the ratio of X chromosomes to sets of autosomes and consequently targeted to the X chromosome in a sex-specific manner; however, inactivation of *dpy-30*, which encodes a subunit of the *C. elegans* dosage compensation complex essential for its recruitment to the X chromosome [45]–[46], did not result in an increase in apoptosis in X0 worms (Table S1), suggesting that a different mechanism distinguishes sex chromosome karyotype in meiosis.

While our study did not reveal the mechanism whereby germ cells distinguish between asynapsed chromosomes and a single X, we have shown that H3K9me2 deposition plays distinct roles in these two situations. In particular, our data demonstrate that H3K9me2 is required for blocking checkpoint signaling and transcriptional silencing on the single X during MSCI but not on asynapsed chromosomes during MSUC (Figure 7). Cytological analyses suggest that there is more robust accumulation of H3K9me2 on a single X chromosome or extra-chromosomal array compared to asynapsed chromosome pairs (see [23]; Figure 5C; Checchi and Engebrecht, unpublished observations), providing a plausible explanation for the differential affect of H3K9me deposition on sequences lacking a pairing partner. Interestingly, studies in mice spermatocytes also suggest that there are more robust chromatin modifications on an unpaired chromosome compared to segmental trisomic regions resulting in asynapsis [47].

Analysis of the histone deacetylase SIN-3, which plays a role in targeting H3K9me2 to unpaired DNA [14], also supports the differential regulation of H3K9me2 on asynapsed homologous chromosomes versus the partnerless X chromosome. Absence of *sin-3* specifically affects H3K9me2 targeting to DNA normally possessing a homologous pairing partner; *him-8* XX germ lines depleted for *sin-3* and carrying a repetitive extra-chromosomal array retain H3K9me2 on the array, yet lack this modification on the asynasped X chromosome pair (Figure S5; Checchi and Engebrecht, unpublished observations). Nonetheless, levels of germline apoptosis in response to chromosome asynapsis are unchanged in *sin-3* mutants (Figure 5A), indicating that SIN-3-dependent deposition of H3K9me2 does not create a chromatin environment that blocks checkpoint signaling. Further, H3K9me2 deposition does not directly mediate the transcriptional silencing that defines MSUC, as the absence of either SIN-3 or MET-2 has no effect on the transcriptional repression of asynapsed chromosome pairs (Figure 5, Figure S5, and Figure 7). Thus, H3K9me2 on the lone X leads to MSCI and checkpoint shielding, whereas this same modification neither mediates transcriptional silencing during MSUC nor modulates checkpoint signaling, suggesting that these processes are distinct.

H3K9me2 deposition in the germ line is also regulated by EGO-1, CSR-1, EKL-1 and DRH-3, components of the small RNA pathway. Mutation of any of these genes results in redistribution of H3K9me2 from both asynapsed chromosomes and the single X of males to paired chromosomes, suggesting that this pathway does not distinguish asynapsed chromosomes from those lacking a pairing partner [41], [48]. Consistent with this, we found that inactivation of these components results in a global increase in apoptotic nuclei in both XX and X0 germ lines (Checchi and Engebrecht, unpublished observations). The effect of this small RNA pathway on transcription has not been examined; thus, the role of this pathway in MSCI and MSUC is unclear.

Although H3K9me2 deposition on asynapsed chromosomes neither blocks transcription nor checkpoint signaling, it does impact fertility. MET-2 plays a subtle role in promoting the fidelity of meiotic chromosome segregation [23] and we observed a progressive sterility defect in both *met-2* and *sin-3* mutants over successive generations (Billadeau and Checchi, unpublished observations). H3K9me2 could facilitate the repair outcome of breaks induced on asynapsed chromosomes by altering chromatin structure and/or preventing inappropriate recombination between non-homologous chromosomes. Another possibility is that H3K9me2 marks asynapsed chromosomes to promote their segregation in the absence of chiasma, thereby improving the likelihood of generating euploid gametes. While the role of H3K9me2 deposition on asynapsed chromosomes has not been elucidated, H3K9me2 is important for genome integrity as the *met-2*; *sin-3* double mutant is sterile. Thus, multiple pathways mediate H3K9me2 deposition and distribution in the germ line and the integration of these pathways in conjunction with the chromosomal context result in distinct regulatory outputs including blocking checkpoint activation and transcription, as well as promoting genome integrity.

### Meiotic checkpoint signaling can occur in the absence of local transcriptional activation

In heterogametic (X0) germ lines depleted for *met-2*, both activated Pol2 and H3K4me2, a mark corresponding to transcriptionally competent chromatin, appear on the X chromosome in late pachytene, coincident with accumulation of ATL-1 (ATR) and elevated levels of apoptosis in these nuclei (Figure 1, Figure 2, Figure 3). Further, heterogametic germ lines depleted for either *met-1* or *mes-2* also exhibit elevated apoptosis as well as ectopic, X chromosome-specific accumulation of H3K4me2 in late pachytene (Table 1; Figure S4). Together, these data suggest that a subset of HMTases specifies a repressive chromatin architecture that blocks checkpoint signaling and precludes acquisition of activating marks and transcription.

While our data do not exclude the possibility that the absence of transcription from the single X is directly responsible for blocking checkpoint signaling in worms, we favor the hypothesis that chromatin architecture directly blocks checkpoint signaling for the following reasons: One, the unmodified form of Pol2 is present on all autosomes but is absent from the single X chromosome, suggesting that the chromatin architecture of the X is inaccessible to protein complexes. Because chromatin accessibility of checkpoint components is critical to signaling [49], the closed chromatin structure of the X is likely to preclude assembly of checkpoint proteins. Two, ATR is not enriched in the nuclei of wild-type male [19] or *fem-3(lf)* X0 germ cells, yet is abundant in nuclei of heterogametic germ lines with asynapsed autosomes (e.g. *zim-1*) (Figure 1B; [19]) even though the X is not transcribed in these germ lines (Figure 3 and Figure 5C). Three, we observed checkpoint-dependent apoptosis in both XX and X0 germ lines depleted or mutant for *him-17* (Table S1; Figure S2C; data not shown), which results in aberrant H3K9me2 accumulation yet normal transcriptional regulation [50], suggesting that alteration in chromatin structure can influence checkpoint signaling without impinging on transcription. Four, we demonstrate that checkpoint response is dependent upon chromatin architecture but not transcription by targeting breaks to the extra-chromosomal *Mos1* substrate array in the absence of *met-2*. In these germ lines H3K9me2 was abrogated and breaks elicited a checkpoint response in the absence of the activating mark H3K4me2 or Pol2 (Figure 6, Figure 7, and Figure S7A-S7B). Together, these results indicate that the checkpoint response to breaks on sequences lacking a partner is dependent upon chromatin architecture but not transcription.

In mice, transcriptional silencing of asynapsed chromosomes or chromosomal regions has been proposed to induce arrest or apoptosis due to silencing of genes essential for meiosis [10]. In *C. elegans*, we provide evidence that transcriptional silencing during MSUC is not absolute, although incomplete silencing may be sufficient to reduce the expression of genes essential for meiosis, thereby inducing apoptosis. The role of transcriptional silencing on checkpoint signaling in the context of MSUC awaits identification of the machinery that blocks transcription on asynapsed chromosomes.

### A complex chromatin environment mediates sex chromosome regulation

Histone modifications (e.g. the “histone code hypothesis”) play an essential role in coordinating numerous cellular processes including the regulation of gene expression, DNA repair and checkpoint signaling [51], [52]. Here, we have uncovered a novel role for MET-2 as well as two additional conserved HMTases, MET-1 and MES-2, in inhibiting X chromosome-specific checkpoint signaling. Inactivation of any one of these HMTases results in elevated apoptosis in worms with a single X chromosome, indicating that no single chromatin mark mediates repression of checkpoint signaling. While MET-2 and MES-2 are required for deposition of the repressive marks enriched on the single unpaired X chromosome, the role of MET-1 is less clear. MET-1 (ortholog of yeast Set2) is a H3K36 HMTase; previous reports indicated that H3K36me corresponds to transcriptional activation, and both yeast and human Set2 directly associate with the elongating form of Pol2 [53], [54]. On the other hand, the mammalian Set2 ortholog Whsc functions in embryonic stem cells as a negative regulator of transcription [55], suggesting that H3K36me can be repressive. Consistent with a repressive role, *C. elegans met-1* was initially identified and characterized as an inhibitor of transcription [21].

In this study we show that like *met-2* and *mes-2* depletion, absence of *met-1* results in an ectopic accumulation of H3K4me2 on the single unpaired X chromosome and elevated germline apoptosis, consistent with a repressive role for MET-1 in this process. *met-1* interacts synthetically with *met-2* as well as *hpl-1/2*, the worm homologs of heterochromatin protein 1 (HP1) during vulval development [21]. Furthermore, the *met-1*; *met-2* double mutant has synthetic effects in the germ line [21], suggesting that a similar repressive pathway may operate to block checkpoint signaling on the unpaired X. While the precise chromatin environment that mediates this process is unclear, we propose that functional interactions between MET-1, MET-2, MES-2, and as yet unidentified factors, are necessary to promote germline homeostasis and facilitate efficient meiotic progression and checkpoint regulation.

### Meiotic behavior of heteromorphic sex chromosomes in worms and mammals

Our data have uncovered a role for repressive chromatin modifiers in mediating both transcriptional silencing and checkpoint shielding of the partnerless X chromosome in *C. elegans*. Sex chromosomes in mammalian males are also subject to transcriptional silencing and as in worms, this appears to be mediated by H3K9me2 deposition [26]. However, whether the orthologous mammalian HMTases play a role in sex chromosome regulation has not been explored. A recent study in mice provides compelling evidence for the importance of MSCI in male fertility [56]; however, as perturbation of both transcriptional silencing and checkpoint signaling results in elevated apoptosis it is difficult to determine the relative contributions of these processes to ensuring the formation of gametes from the heterogametic sex. Our analyses in *C. elegans* suggest that the epigenetic landscape of sex chromosomes is complex and plays a critical role in ensuring transmission of sex chromosome through meiosis through both transcriptional silencing and checkpoint shielding.

## Materials and Methods

### Alleles and strain maintenance

Maintenance and genetic analysis of worms were performed using standard procedures [57]. *C. elegans* var. Bristol (N2) was used as the wild-type strain. The following mutations were used in this study: LG1, *sin-3(tm1276)*, *cep-1(gk138)*, *met-1(n4337)*; LGII, *pch-2(tm1458)*; LGIII, *met-2(n4256)*; LGIV, *fem-3(e1996)/nT1-GFP*, *him-8(me4)*, *zim-1(tm1813)*; *zim-2(tm574)*; LGV, *him-17(e2806)*; LGX, *lon-2(e678)*. Some nematode strains used in this work were provided by the *Caenorhabditis* Genetics Center, which is funded by the National Institutes of Health National Center for Research Resources (NCRR). Homozygous X0 female worms were generated as described in [13]. Transgenic strains containing the *Mos1* template array (*oxEx229*) and the HSP:*Mos1*-transposase array (*oxEx166*) were used for *Mos1* mutagenesis experiments [58]. All strains were propagated at 20°C, unless otherwise noted.

### Quantification of germline apoptosis

Apoptotic germline nuclei were scored in indicated worms approximately 48 hrs post L4 larvae by acridine orange (AO) or CED-1::GFP [24] as described in [13].

### Immunofluorescence analysis

Immunostaining of germ lines was performed as described in [59] except anti-Pol2 CTD (8WG16) staining where whole-mount gonads were fixed in 5% formaldehyde, followed by a 2 min. post-fix in −20°C 95% ethanol prior to washing and antibody addition. Guinea pig anti-HIM-8 (1∶500) and rabbit anti-ATL-1 (1∶500) were generous gifts from Abby Dernburg and Simon Boulton, respectively. The following primary antibodies were purchased and used at the indicated dilutions: rabbit anti-histone H3K9me2 and rabbit-anti-histone H3K27me3, 1∶500 (Millipore; Billerica, MA), rabbit anti-histone H3K4me2, 1∶500 (Cell Signaling Technology; Danvers, MA), mouse anti-histone H3K9me2 and rabbit-anti-histone H3K9me3, 1∶500 (AbCam; Cambridge, MA), rabbit anti-HIM-8, 1∶500 (SDIX; Newark, DE), rabbit-anti-phospho-Chk1(Ser345), 1∶50 (Santa Cruz Biotechnology, Inc.; Santa Cruz, CA), rabbit-anti-RAD-51, 1∶1000 (Novus Biologicals; Littleton, CO) and rabbit anti-GFP, 1∶500 (Novus Biologicals; Littleton, CO). To detect the phosphorylated or unphosphorylated form of RNA Pol II, the monoclonal antibodies H14 (Pol2 Ser5-P), 1∶50 and 8WG16 (Pol2 CTD), 1∶500 (Covance; Princeton, NJ) were used, respectively. DyLight™649 donkey anti-guinea pig IgG (Jackson ImmunoResearch Laboratories; West Grove, PA) and the following secondary antibodies from Molecular Probes® (Invitrogen; Carlsbad, California) were all used at 1∶500 dilutions: Alexa Fluor® 546 goat anti-mouse IgG, Alexa Fluor® 488 goat anti-mouse IgG, Alexa Fluor® 555 goat anti-rabbit IgG, Alexa Fluor® 488 goat anti-rabbit IgG, Alexa Fluor® 488 goat anti-guinea pig IgG. DAPI (Sigma, 1 µg/ml) was used to counterstain DNA. Collection of images was performed using an API Delta Vision deconvolution microscope. Images were deconvolved using Applied Precision SoftWoRx image analysis software and were subsequently processed and analyzed using ImageJ (Wayne Rasband, NIH). All images shown are projections through data stacks.

### RNA–mediated interference (RNAi) analysis

All RNAi experiments in this study were performed at 20°C, using the feeding method, as described in [60]. L4 larvae were fed RNAi-inducing HT115(DE3) bacteria strains from an available RNAi feeding library [61]. As a control, worms were fed the same bacteria, except that it was transformed with the empty RNAi feeding vector, L4440. RNAi constructs not available from the feeding library (e.g. *him-17* and *mes-2*) were cloned from a worm cDNA library into L4440. Cultures were plated onto NGM plates containing 25 µg/ml Carbenicillin and 1 mM IPTG. For double RNAi experiments (see Figure 1A), parents were mated on either control (L4440) or *met-2*(RNAi) plates and F1 *fem-3*(lf) X0 progeny were picked as L4s and transferred to either L4440, *pch-2*(RNAi) or *cep-1*(RNAi) plates for 48 hours before scoring.

### *Mos1*-mediated mutagenesis

*Mos1*-mediated mutagenesis was performed using the protocol described in [58]. Targeted DSBs were induced by expressing *Mos1* transposase, encoded on an extra-chromosomal array (*oxEx166*) whose expression is regulated by a heat shock-inducible promoter. To examine the consequence of DSBs targeted to an extra-chromosomal *Mos1* template array (*oxEx229*), double-transgenic worms (*oxEx166*; *oxEx229*) were generated and propagated for one generation on either OP50 or RNAi feeding plates. Efficiency of *Mos1* transposition was determined by PCR amplification of the *Mos1* sequence in the offspring of heat-shocked worms, as described in Boulin and Bessereau [58]; at least one *Mos1* insertion was present in ≥80% of animals tested (data not shown).

## Supporting Information

Figure S1 RNAi depletion of *met-2* disrupts H3K9me2 deposition in the heterogametic (X0) germ line. Immunolocalization of H3K9me2 (red) in pachytene *fem-3(lf)* X0 germ lines fed empty L4440 vector or *met-2* dsRNA (left) and *met-2(n4256)* and *met-1(n4337)* male germ lines (right). Germ lines were counterstained with DAPI (blue). Scale bar = 10 µm.
(TIFF)

Figure S2 Apoptotic bodies in XX versus X0 mutant germ lines. (A) Cytological analysis of control (L4440) *fem-3(lf)* X0 (top) versus *met-2*(RNAi); *fem-3(lf)* X0 (bottom) germ lines expressing CED-1::GFP. Germ lines were stained with anti-GFP (red) and counterstained with DAPI (blue). White arrowheads denote CED-1::GFP(+) nuclei. Yellow arrows indicate late-stage corpses. Scale bar = 10 µm. (B) Number apoptotic bodies in XX (left) versus *fem-3(lf)* X0 (right) germ lines as determined by CED-1::GFP fluorescence approx. 48 hr post L4 stage. Total number of gonads examined: N2 XX L4440, *N* = 296; *met-2*(RNAi) XX, *N* = 123; *met-2(n4256)* XX, *N* = 58; *fem-3(lf)* X0, *N* = 211; *met-2*(RNAi);*fem-3(lf)* X0, *N* = 85. (C) Number of apoptotic nuclei per gonad arm measured by acridine orange (AO) staining approx. 48 hr post L4 stage. Total number of gonads examined: N2 XX, *N* = 41; *met-2(n4256)* XX, *N* = 35; *met-1(n4337)*, *N* = 38; *him-17(e2806)*, *N* = 27; *zim-2(tm574)*, *N* = 17. Statistical comparisons between mutants were conducted using the two-tailed Mann-Whitney test. * denotes p≤0.001. Error bars correspond to S.E.M.
(TIFF)

Figure S3 *met-2* does not affect the accumulation or processing of X chromosome-specific DSBs. (A) Immunolocalization of H3K9me2 (red) and RAD-51 (green) in a wild-type male germ line. Red asterisk denotes distal tip. Scale bar = 10 µm. (B) Histograms comparing quantification of RAD-51 foci on the wild-type male X chromosome (left) and the *met-2(n4256)* male X chromosome (right). Y axis indicates the percentage of nuclei that contained 0, 1 or 2–3 RAD-51 foci during early pachytene (light gray) or mid/late pachytene (dark gray). RAD-51 foci were quantitated as described in [13]. Total number of nuclei scored: wild-type early pachytene, *N* = 101; wild-type late pachytene, *N* = 82; *met-2* early pachytene, *N* = 97; *met-2* late pachytene, *N* = 101. No RAD-51 foci were observed in *spo-11* mutants, indicating specificity of antibody. (C) Histograms comparing quantification of RAD-51 foci on the X chromosome in pachytene stage *rad-54*(RNAi) male germ line nuclei (white; *N* = 48) versus *met-2*;*rad-54*(RNAi) germ line nuclei (black; *N* = 49). *rad-54* depletion was assessed by progeny inviability.
(TIFF)

Figure S4 H3K4me2 accumulates ectopically on the single X chromosome in X0 germ lines depleted for *met-1* or *mes-2*. Immunolocalization of H3K4me2 (red) counterstained with DAPI (blue) in *fem-3(lf)* X0 germ lines fed (A) *met-1* dsRNA (left) or (B) *mes-2* dsRNA (right). Green outline indicates the X chromosome in mid-pachytene (MP), late pachytene (LP), and LP/diplotene (DP), as determined by HIM-8 staining (green). White arrows indicate the X chromosome in DP and diakinesis (DI). Scale bar = 5 µm. (See also Figure 2).
(TIFF)

Figure S5 SIN-3 targets H3K9me2 to asynapsed chromosome pairs but not to a repetitive extra-chromosomal array. (A) Immunolocalization of H3K9me2 (red) in wild-type N2 XX (top), *sin-3*(RNAi) XX (middle), and *met-2*(RNAi) XX (bottom) in mid-pachytene germ lines carrying the extra-chromosomal array *oxEx229*. Scale bar = 10 µm. Inset: A single nucleus (indicated in main panel by white box) accumulates H3K9me2 on the array in wild-type and *sin-3*(RNAi) germ lines (top and middle) but lacks this mark in the absence of *met-2* (bottom). Red outlines indicate the extra-chromosomal array as determined by size/chromatin condensation. Scale bar = 1 µm. (B) *him-8(me4)* XX mutants fed either empty L4440 vector (top) or *sin-3* dsRNA (bottom) were stained with H3K9me2 (red) and HIM-8 (green) and were counterstained for DAPI (blue). Insets show individual mid-pachytene stage nuclei. White arrowheads denote unpaired X chromosomes (identified by HIM-8, green). Scale bar = 10 µm.
(TIFF)

Figure S6 In *C. elegans*, MSUC corresponds to transcriptional inactivation of asynapsed chromosome pairs that is independent from H3K9me2 deposition. (A) *him-8(me-4)*, *zim-1(tm1813)*, and *zim-2(tm574)* XX germ lines stained with Pol2 Ser5-P (red) and counterstained with DAPI (blue). In *him-8(me4)* XX germ lines, the asynapsed chromosomes were identified by co-staining with HIM-8 (green). Inset: An asynapsed late pachytene chromosome pair (indicated by white arrowheads) lacks Pol2 Ser5-P staining. Scale bar = 10 µm. (B) Whole-mount *him-8(me-4)* XX germ line stained with Pol2 Ser5-P (red) and H3K9me2 (green) and were counterstained with DAPI (blue). White arrow (in merge) indicates direction of meiotic progression. Boxed section corresponds to inset. Scale bar = 10 µm. Inset: Most late pachytene nuclei lack Pol2 Ser5-P staining on the asynapsed X chromosome pairs (indicated by white arrowheads), but this does not always correspond to H3K9me2 deposition. Scale bar = 5 µm. (C) *met-2*(RNAi);*him-8(me-4)* XX pachytene nuclei stained with Pol2 Ser5-P and H3K9me2 (green) and counterstained with DAPI (red) are completely devoid H3K9me2 yet do not affect transcription on the asynapsed X chromosomes. Scale bar = 5 µm.
(TIFF)

Figure S7 On an unpaired, extra-chromosomal array, absence of MET-2 does not affect H3K4me2 and H3K9me3 dynamics. (A–B) Immunolocalization of H3K4me2 (green) and H3K9me2 (blue) in wild-type XX germline nuclei (top panels) versus *met-2*(RNAi) XX nuclei (bottom panels) containing the extra-chromosomal array *oxEx229*. Germ lines were counterstained with DAPI (red). White arrows correspond to *oxEx229* in late pachytene nuclei (A) and diakinesis nuclei (B). Array was identified by size/chromatin condensation (A–C). (C) Immunolocalization of H3K9me3 (red) in wild-type XX germ lines (top) and *met-2*(RNAi) XX germ lines (bottom) carrying *oxEx229*. Germ lines were counterstained with DAPI (blue). White arrows correspond to *oxEx229* in late pachytene stage nuclei. Mid-pachytene (MP); Late pachytene (LP); diplotene (DP); diakinesis (DI). Scale bar = 5 µm.
(TIFF)

Table S1 Candidate genes surveyed for CED-1::GFP(+) nuclei. Apoptotic bodies were scored by quantifying CED-1::GFP expressing nuclei per gonad arm. A minimum of 24 gonad arms was scored for each genotype. Animals were screened by picking either L4 wild-type XX hermaphrodites or young adult *fem-3(lf)* X0 females expressing CED-1::GFP to RNAi feeding plates (see Materials and Methods) and scored after approx. 48 hrs. *syp-1*(RNAi) and *him-8*(RNAi) were used as positive controls in *fem-3(lf)* X0 and wild-type XX germ lines, respectively. Data shown are means ± S.E.M., and statistical comparisons between RNAi knockdown and empty L4440 vector were determined using a two-tailed Mann-Whitney test; ** denotes p<0.05; * denotes p<0.001. Abbreviations: SC, synaptonemal complex; CO, crossover; PC, pairing center; HP, heterochromatin protein; HAT, histone acetyltransferase; HDAC, histone deacetylase; HMT, histone methyltransferase; HDMT, histone demethylase.
(XLS)
