# Supplementary figures and images for: Caenorhabditis elegans Histone Methyltransferase MET-2 Shields the Male X Chromosome from Checkpoint Machinery and Mediates Meiotic Sex Chromosome Inactivation
Source: PLoS Genet. 2011 Sep 1;7(9):e1002267. doi: 10.1371/journal.pgen.1002267 (PMC3164706; doi:10.1371/journal.pgen.1002267)

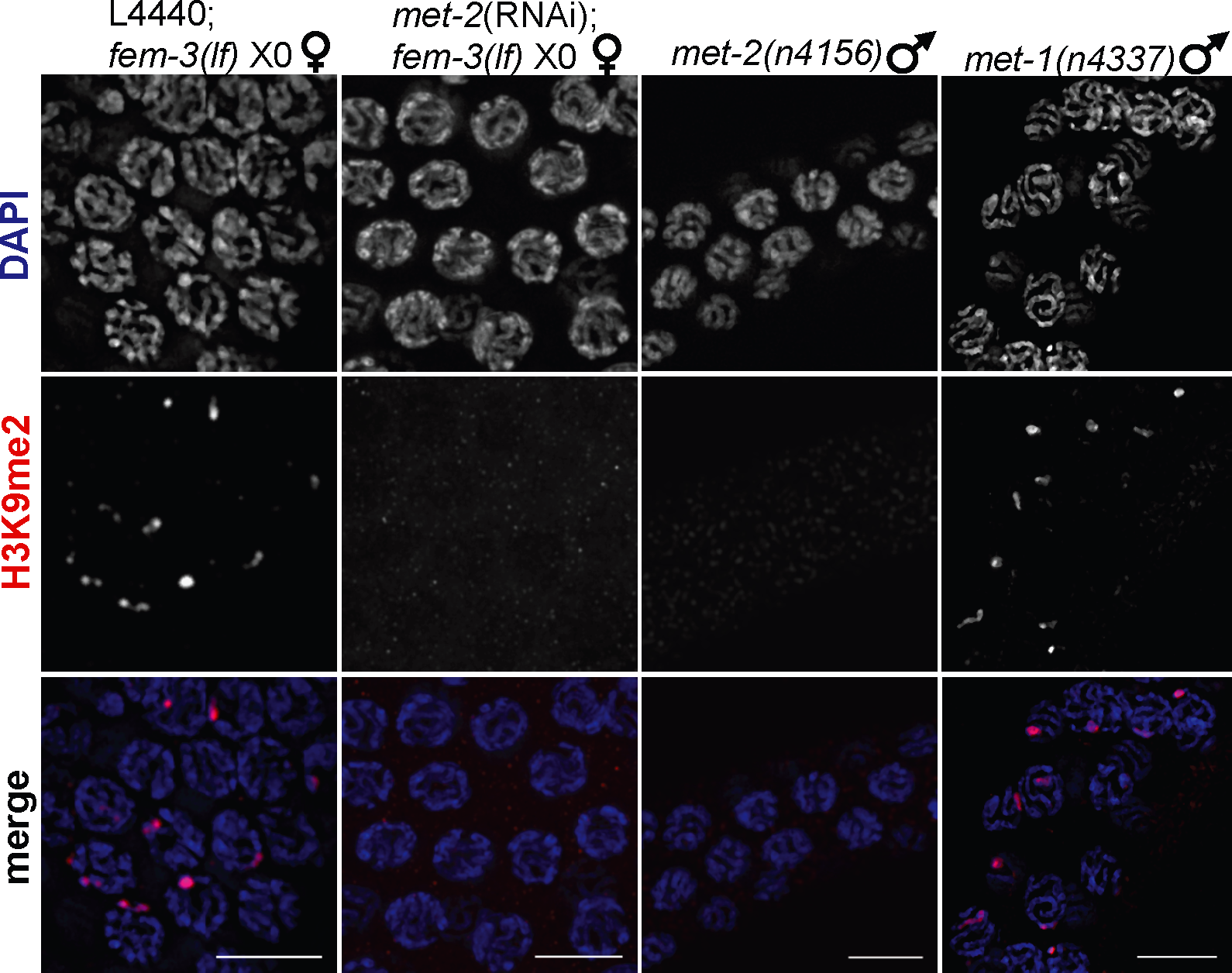

Supplement: Figure S1 — RNAi depletion of met-2 disrupts H3K9me2 deposition in the heterogametic (X0) germ line. Immunolocalization of H3K9me2 (red) in pachytene fem-3(lf) X0 germ lines fed empty L4440 vector or met-2 dsRNA (left) and met-2(n4256) and met-1(n4337) male germ lines (right). Germ lines were counterstained with DAPI (blue). Scale bar = 10 µm. (TIFF) [file pgen.1002267.s001.tiff]

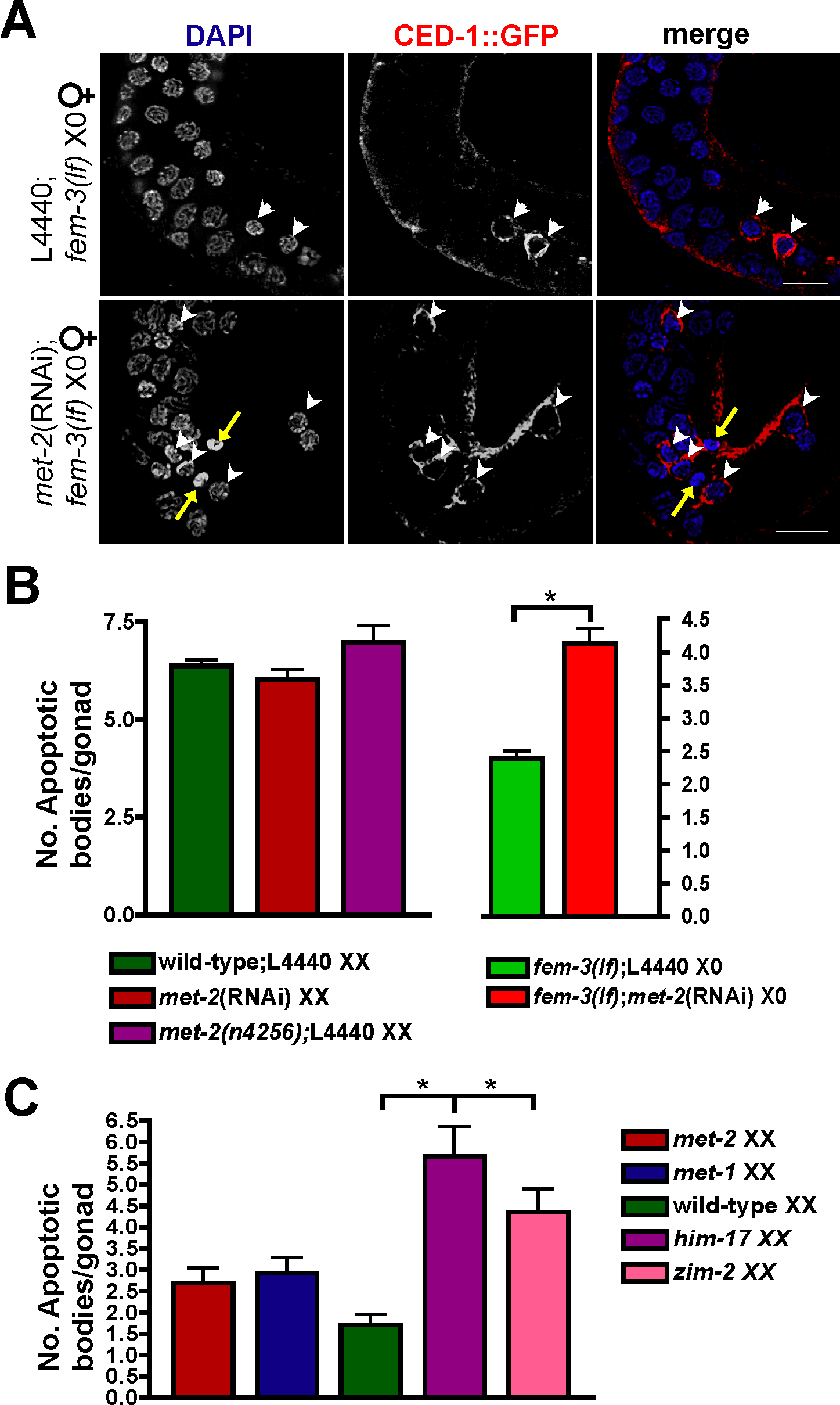

Supplement: Figure S2 — Apoptotic bodies in XX versus X0 mutant germ lines. (A) Cytological analysis of control (L4440) fem-3(lf) X0 (top) versus met-2(RNAi); fem-3(lf) X0 (bottom) germ lines expressing CED-1::GFP. Germ lines were stained with anti-GFP (red) and counterstained with DAPI (blue). White arrowheads denote CED-1::GFP(+) nuclei. Yellow arrows indicate late-stage corpses. Scale bar = 10 µm. (B) Number apoptotic bodies in XX (left) versus fem-3(lf) X0 (right) germ lines as determined by CED-1::GFP fluorescence approx. 48 hr post L4 stage. Total number of gonads examined: N2 XX L4440, N = 296; met-2(RNAi) XX, N = 123; met-2(n4256) XX, N = 58; fem-3(lf) X0, N = 211; met-2(RNAi);fem-3(lf) X0, N = 85. (C) Number of apoptotic nuclei per gonad arm measured by acridine orange (AO) staining approx. 48 hr post L4 stage. Total number of gonads examined: N2 XX, N = 41; met-2(n4256) XX, N = 35; met-1(n4337), N = 38; him-17(e2806), N = 27; zim-2(tm574), N = 17. Statistical comparisons between mutants were conducted using the two-tailed Mann-Whitney test. * denotes p≤0.001. Error bars correspond to S.E.M. (TIFF) [file pgen.1002267.s002.tiff]

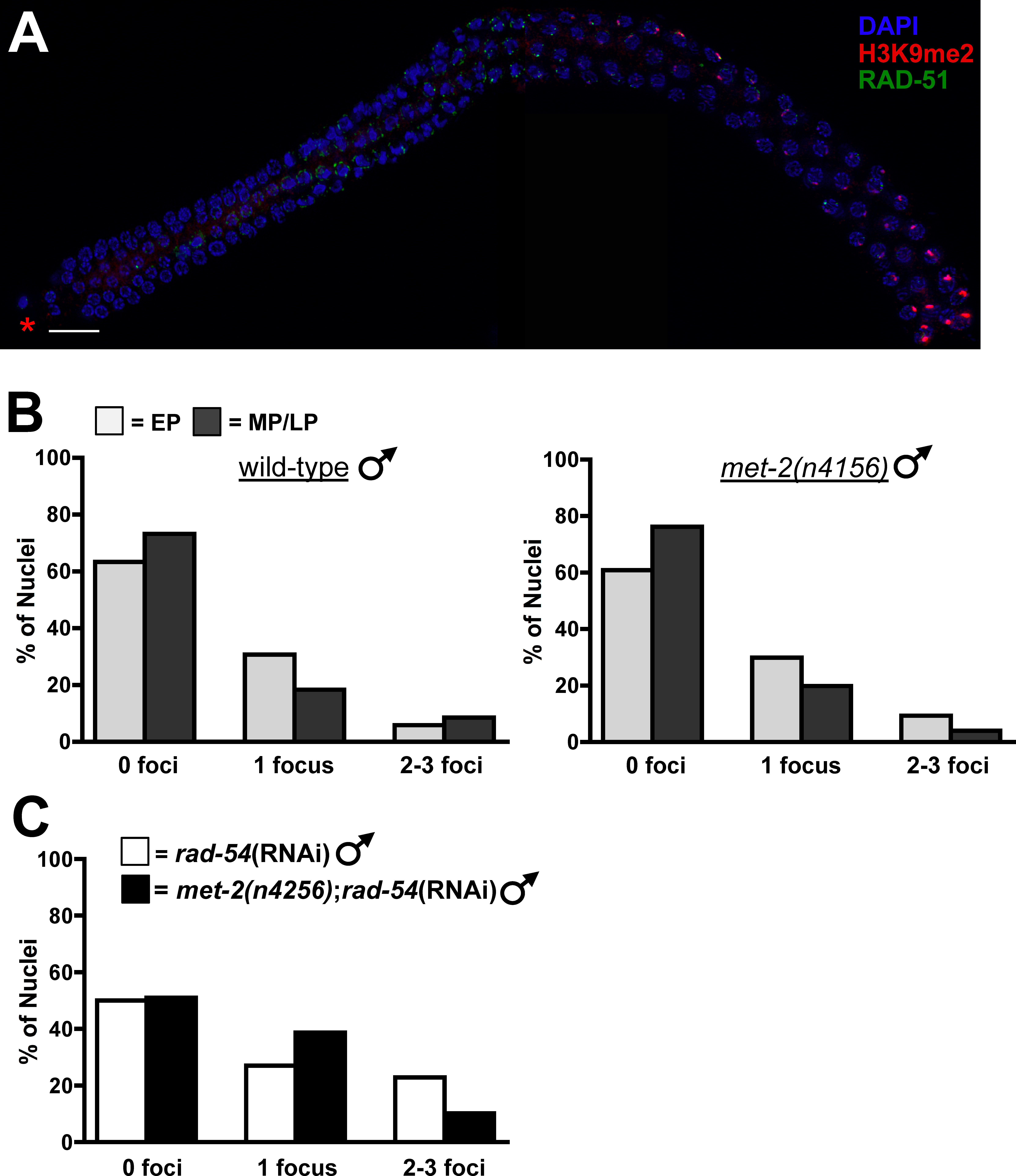

Supplement: Figure S3 — met-2 does not affect the accumulation or processing of X chromosome-specific DSBs. (A) Immunolocalization of H3K9me2 (red) and RAD-51 (green) in a wild-type male germ line. Red asterisk denotes distal tip. Scale bar = 10 µm. (B) Histograms comparing quantification of RAD-51 foci on the wild-type male X chromosome (left) and the met-2(n4256) male X chromosome (right). Y axis indicates the percentage of nuclei that contained 0, 1 or 2–3 RAD-51 foci during early pachytene (light gray) or mid/late pachytene (dark gray). RAD-51 foci were quantitated as described in [13]. Total number of nuclei scored: wild-type early pachytene, N = 101; wild-type late pachytene, N = 82; met-2 early pachytene, N = 97; met-2 late pachytene, N = 101. No RAD-51 foci were observed in spo-11 mutants, indicating specificity of antibody. (C) Histograms comparing quantification of RAD-51 foci on the X chromosome in pachytene stage rad-54(RNAi) male germ line nuclei (white; N = 48) versus met-2;rad-54(RNAi) germ line nuclei (black; N = 49). rad-54 depletion was assessed by progeny inviability. (TIFF) [file pgen.1002267.s003.tiff]

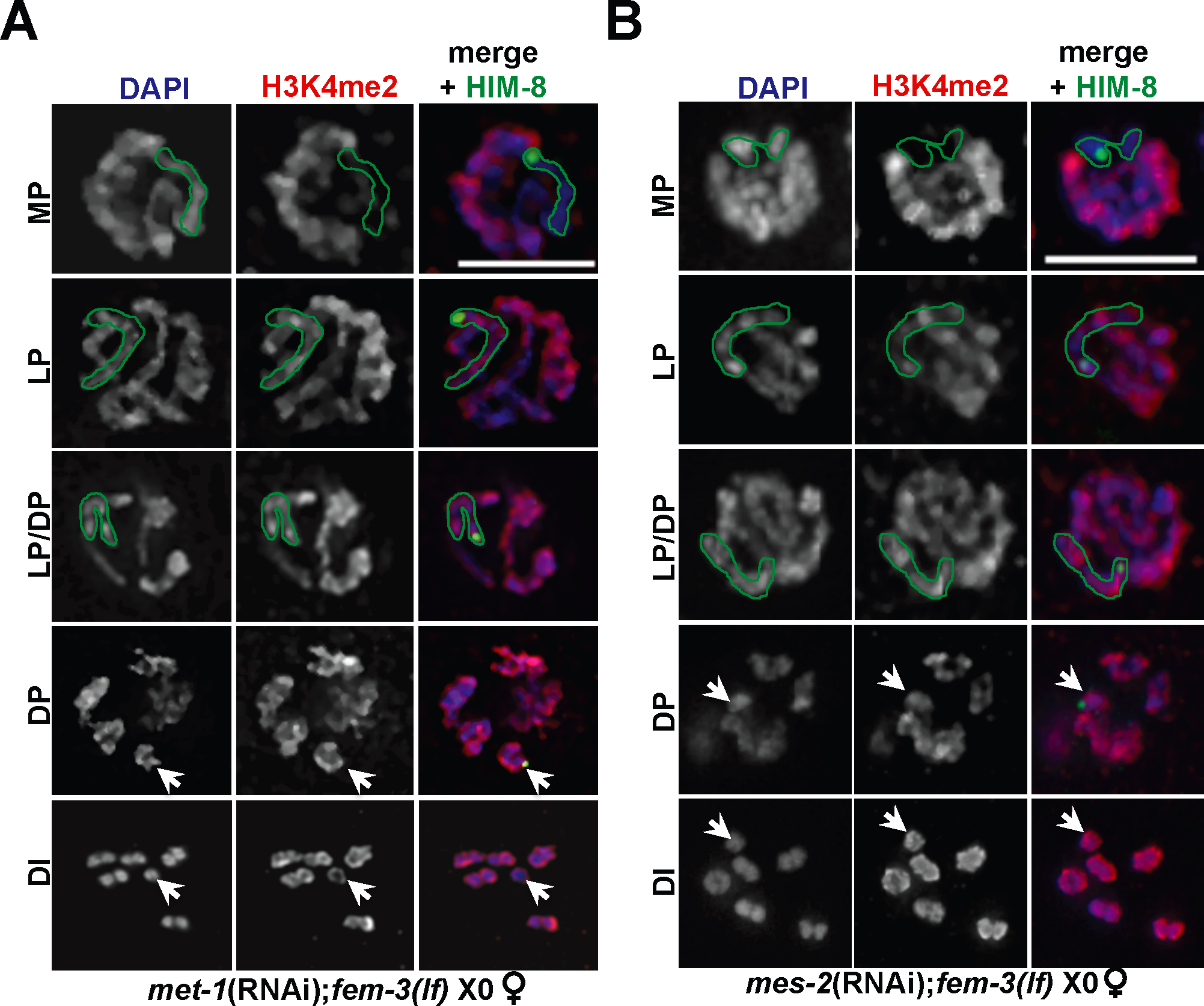

Supplement: Figure S4 — H3K4me2 accumulates ectopically on the single X chromosome in X0 germ lines depleted for met-1 or mes-2. Immunolocalization of H3K4me2 (red) counterstained with DAPI (blue) in fem-3(lf) X0 germ lines fed (A) met-1 dsRNA (left) or (B) mes-2 dsRNA (right). Green outline indicates the X chromosome in mid-pachytene (MP), late pachytene (LP), and LP/diplotene (DP), as determined by HIM-8 staining (green). White arrows indicate the X chromosome in DP and diakinesis (DI). Scale bar = 5 µm. (See also Figure 2). (TIFF) [file pgen.1002267.s004.tiff]

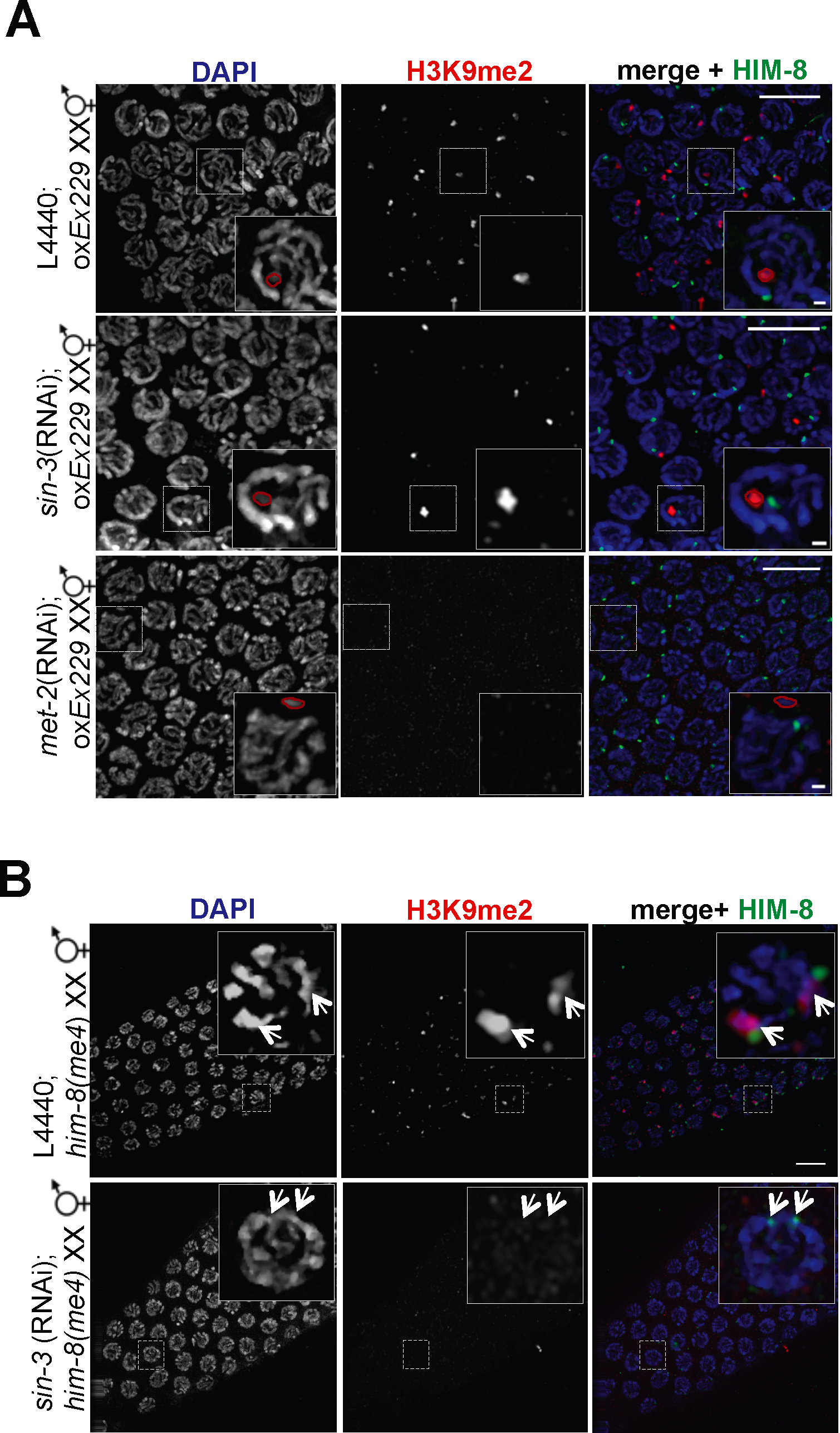

Supplement: Figure S5 — SIN-3 targets H3K9me2 to asynapsed chromosome pairs but not to a repetitive extra-chromosomal array. (A) Immunolocalization of H3K9me2 (red) in wild-type N2 XX (top), sin-3(RNAi) XX (middle), and met-2(RNAi) XX (bottom) in mid-pachytene germ lines carrying the extra-chromosomal array oxEx229. Scale bar = 10 µm. Inset: A single nucleus (indicated in main panel by white box) accumulates H3K9me2 on the array in wild-type and sin-3(RNAi) germ lines (top and middle) but lacks this mark in the absence of met-2 (bottom). Red outlines indicate the extra-chromosomal array as determined by size/chromatin condensation. Scale bar = 1 µm. (B) him-8(me4) XX mutants fed either empty L4440 vector (top) or sin-3 dsRNA (bottom) were stained with H3K9me2 (red) and HIM-8 (green) and were counterstained for DAPI (blue). Insets show individual mid-pachytene stage nuclei. White arrowheads denote unpaired X chromosomes (identified by HIM-8, green). Scale bar = 10 µm. (TIFF) [file pgen.1002267.s005.tiff]

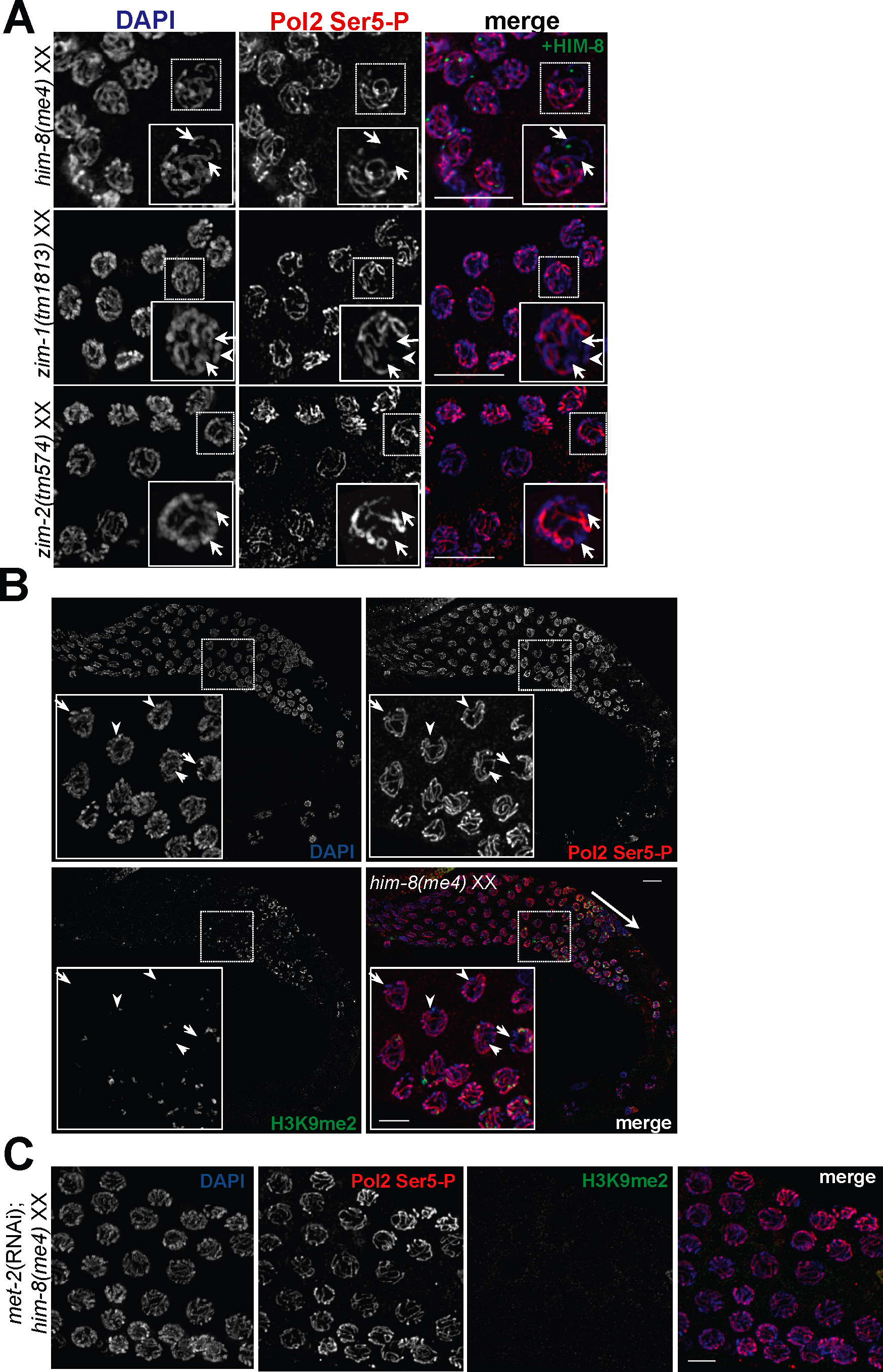

Supplement: Figure S6 — In C. elegans, MSUC corresponds to transcriptional inactivation of asynapsed chromosome pairs that is independent from H3K9me2 deposition. (A) him-8(me-4), zim-1(tm1813), and zim-2(tm574) XX germ lines stained with Pol2 Ser5-P (red) and counterstained with DAPI (blue). In him-8(me4) XX germ lines, the asynapsed chromosomes were identified by co-staining with HIM-8 (green). Inset: An asynapsed late pachytene chromosome pair (indicated by white arrowheads) lacks Pol2 Ser5-P staining. Scale bar = 10 µm. (B) Whole-mount him-8(me-4) XX germ line stained with Pol2 Ser5-P (red) and H3K9me2 (green) and were counterstained with DAPI (blue). White arrow (in merge) indicates direction of meiotic progression. Boxed section corresponds to inset. Scale bar = 10 µm. Inset: Most late pachytene nuclei lack Pol2 Ser5-P staining on the asynapsed X chromosome pairs (indicated by white arrowheads), but this does not always correspond to H3K9me2 deposition. Scale bar = 5 µm. (C) met-2(RNAi);him-8(me-4) XX pachytene nuclei stained with Pol2 Ser5-P and H3K9me2 (green) and counterstained with DAPI (red) are completely devoid H3K9me2 yet do not affect transcription on the asynapsed X chromosomes. Scale bar = 5 µm. (TIFF) [file pgen.1002267.s006.tiff]

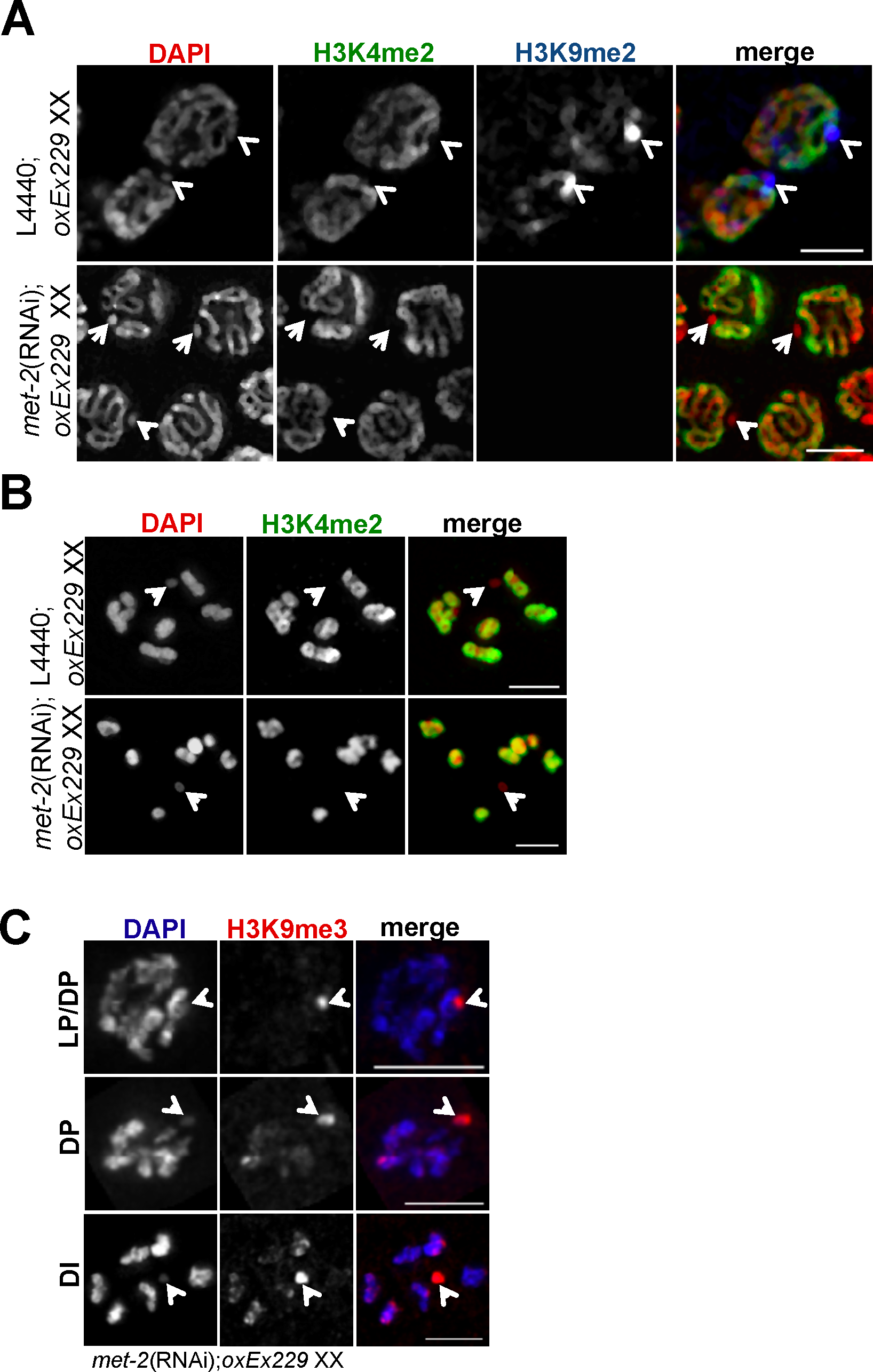

Supplement: Figure S7 — On an unpaired, extra-chromosomal array, absence of MET-2 does not affect H3K4me2 and H3K9me3 dynamics. (A–B) Immunolocalization of H3K4me2 (green) and H3K9me2 (blue) in wild-type XX germline nuclei (top panels) versus met-2(RNAi) XX nuclei (bottom panels) containing the extra-chromosomal array oxEx229. Germ lines were counterstained with DAPI (red). White arrows correspond to oxEx229 in late pachytene nuclei (A) and diakinesis nuclei (B). Array was identified by size/chromatin condensation (A–C). (C) Immunolocalization of H3K9me3 (red) in wild-type XX germ lines (top) and met-2(RNAi) XX germ lines (bottom) carrying oxEx229. Germ lines were counterstained with DAPI (blue). White arrows correspond to oxEx229 in late pachytene stage nuclei. Mid-pachytene (MP); Late pachytene (LP); diplotene (DP); diakinesis (DI). Scale bar = 5 µm. (TIFF) [file pgen.1002267.s007.tiff]
